# Interfacial nano-engineering of MXene-integrated carbon fiber composites for safer type V hydrogen tanks

**DOI:** 10.1039/d6ra04644d

**Published:** 2026-07-21

**Authors:** Ayyaz Ali Janjua, Oludare Amos Solademi, Emmanuel Okechukwu Achukwu, Nadimul Haque Faisal, Mohd Shahneel Saharudin

**Affiliations:** a School of Computing, Engineering and Technology, Robert Gordon University Garthdee Road Aberdeen AB10 7GJ UK a.janjua@rgu.ac.uk s.saharudin@rgu.ac.uk; b Department of Polymer and Texile Engineering, Ahmadu Bello University Zaria Nigeria; c Universiti Kuala Lumpur, Malaysia Italy Design Institute 119 Jalan 7/91, Taman Shamelin Perkasa Kuala Lumpur 56100 Malaysia

## Abstract

The fabrication of Type V composite overwrapped pressure vessels (COPVs) for compressed hydrogen storage necessitates comprehensive material-level understanding of how fibre orientation and nanofiller concentration jointly determine structural integrity, gas-barrier performance and thermal stability. This research investigates MXene-integrated unidirectional (UD) and woven carbon fibre/epoxy composites as promising materials for Type V COPV manufacturing through a unified framework combining laminate fabrication, energy-dispersive spectroscopy (EDS), thermogravimetric analysis (TGA), differential scanning calorimetry (DSC), tensile testing, scanning electron microscopy (SEM), non-destructive evaluation (NDE), and finite element modelling (FEM). The incorporation of MXene nanoflakes at a loading of 0.5 wt% yielded peak improvements of 18.39% and 22.07% in tensile strength for UD and twill laminates, respectively, while stiffness measurements emphasized a concentration-mediated effect ascribed to MXene agglomerations at higher loadings. TGA and DSC confirmed enhanced thermal stability with a 10 °C increment in glass transition temperature, demonstrating an increased degree of crosslinking. Hydrogen permeation testing through MXene-integrated UD laminates achieved a steady-state diffusion and sorption stage after repeated pressurization events as the permeation values varied marginally between 18.2 and 18.8 mL m^−2^ day^−1^ atm^−1^, confirming a converged steady-state condition. Linear regression achieved *R*^2^ values of 0.9617 and 0.84 for tensile strength and stiffness, respectively. MXene-incorporated UD composites highlighted their capability to avoid early leakage and achieved an equilibrium diffusion state under pressurized loading, suggesting their potential to carry load and serve as hydrogen barrier materials, an essential requirement for linerless Type V hydrogen storage pressure vessels.

## Abstract 2

Optimizing fibre orientation and MXene content improves the structural integrity, hydrogen barrier, and thermal stability of Type V COPVs.

## Graphical abstract

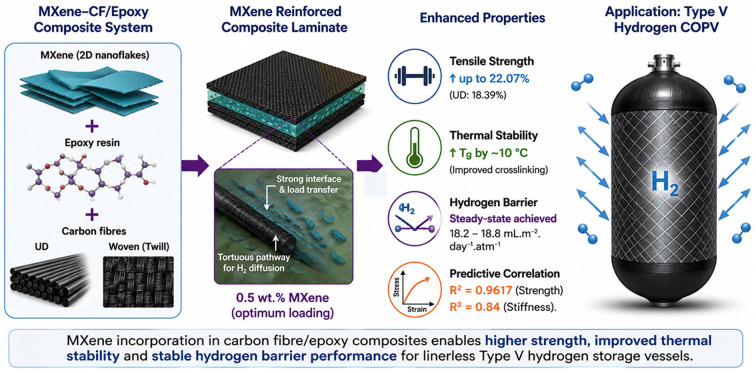

## 1 Introduction

A commonly adopted practice to store gases under high pressure for aerospace and automotive applications is within carbon fibre overwrapped pressure vessels.^1^ Composite overwrapped pressure vessels are better options to store gases^2^ as the interest in utilizing hydrogen as a low carbon fuel is rapidly increasing.^3^ Several types of composite overwrapped pressure vessels are available in the market; these are categorized as Types II, III, and IV. These vessels consist of two layers: an internal liner that holds the liquid or gas and an outer layer composed of a high-strength fibre material that provides reinforcement. The liners are generally made of a thin metal or a thermoplastic polymer, which function as a sealed containment layer, while the high-strength composite overwrap shields the liner material from external damage and holds the structural integrity of the vessel.^4,5^ However, linerless Type V COPVs highlight the latest advancement in gas storage; their full operational capabilities are yet to be explored.^6,7^ Type V pressure vessels are commonly known as composite pressure vessels (CPVs), which offer notable benefits compared to Type IV in terms of weight reduction^8^ and^9–11^ increased volumetric capacity and by promoting gas leakage before burst failure.^12^

Despite its engineering challenges, hydrogen storage optimisation offers a pathway to reduce the reliance on fossil fuels and markedly lower carbon emissions that would aid global efforts towards decarbonisation. Traditional energy sources, like oil, coal, and natural gas, release carbon dioxide and other harmful byproducts upon combustion, accelerating environmental degradation and global warming.^13–15^ Moreover, these natural resources are not renewable and will be depleted eventually.^16,17^ On the other hand, although renewable sources are alternate sources of energy, their intermittent, seasonal, and variable nature often leads to imbalances between energy supply and demand.^18^ Hydrogen has emerged as a technically sound replacement for natural fuels and is widely accepted as the future energy carrier.^19^ Hydrogen holds the highest specific energy, and it releases water as a byproduct, representing an environmentally sustainable alternative to conventional fuels. Due to its abundance, hydrogen provides a promising solution to meet high global energy demands. At ambient pressure, hydrogen has low volumetric energy and density and requires a substantial storage capacity in its gaseous phase. To overcome these challenges and to attain an efficient operational range for transport applications, hydrogen is normally compressed to high pressures.^20^

A lot of research on composite overwrapped pressure vessels has been published in the literature, covering various aspects from their design and development to their decommissioning. Composite-wrapped pressure vessels display more complex damage modes compared to traditional pressure vessels. On a micro-dimensional scale, the damage in composite materials involves fibre breakage, matrix cracking and debonding. These damage mechanisms can compromise vessel strength, leak-before-burst behaviour, and operational lifespan, especially under high-pressure cycling.^21–24^ With the use of composite pressure vessels continuously extending, concerns related to their safety and mechanical integrity have grown. Damage arises from various factors, including manufacturing defects, impact events, fatigue, and operational service conditions.^21,23,25^ Various non-destructive structural health monitoring techniques have been developed to detect damage within composite overwrapped pressure vessels, including X-ray radiography,^26^ acoustic emissions,^27^ optical fibre sensing,^28^ and digital image correlation.^29^ Damage detection and diagnosis within composite overwrapped pressure vessels have proved to be challenging due to their complex anisotropic structure. The propagation of guided waves through composite vessels is affected by numerous factors, including material anisotropy,^30^ thickness,^31^ and curvature;^32^ all these influence the reliability and accuracy of the results. In recently published papers on composite overwrapped pressure vessels (COPVs), many researchers have focused on the fabrication and test performance of Type V linerless vessels. These vessels are subjected to intensive burst testing, typically at 350 to 700 bar pressure, to assess their strength and feasibility for high-pressure deployments, such as hydrogen storage in the aerospace and automotive sectors. These tests assist in identifying the tolerance of vessels to rupture stress and fatigue, predominantly after enduring autofrettage, a procedure developed to improve their resilience under operational conditions.^33,34^

Researchers have documented numerous studies on composite overwrapped pressure vessels in the literature, spanning multiple areas from design to fabrication to their operational use and eventually end-of-life management. These studies provided significant information on material selection, performance under pressure, and long-term reliability under varying environments. Perillo *et al.*^35^ quantified the residual burst strength of composite pressure vessels following mechanical impacts, establishing a correlation between burst pressure and the energy of the impact. Similarly, Blanc-Vannet's^19^ investigation into the effects of impact and post-impact compression response of single stiffened composite samples demonstrated that the use of fillers significantly enhances load-bearing capacity and improves damage tolerance. These studies provide critical insights into the structural resilience and failure mechanisms of composite vessels under impact conditions.

The global climate crisis, which is marked by concerns over ozone layer depletion, global warming, and air and water pollution caused by greenhouse gas emissions, in particular carbon dioxide and methane, has accelerated efforts toward achieving carbon neutrality and a net-zero economy. This has driven a significant reduction in reliance on fossil-fuel-based energy sources and led to a sharp increase in the adoption of renewable energy vectors to facilitate the transition from fossil-based to renewable energy systems.^36,37^ In recent years, hydrogen storage in pressure vessels has increasingly required pressures of up to 70 MPa, particularly for applications in the transportation sector. This has heightened the importance of minimizing both the weight and cost of these vessels to enhance efficiency and feasibility in commercial and industrial use.^38^ Hydrogen storage presents a significant challenge due to its high energy content by weight but low energy density by volume, as highlighted by Reynolds *et al.*^39^ and Curtis *et al.*^36^ This discrepancy complicates efficient storage solutions, particularly when aiming to store large volumes of hydrogen in compact spaces for industrial and transportation applications. One of the key challenges to the widespread adoption and use of hydrogen as an energy carrier is the development of reliable, safe, compact, and cost-effective storage technologies.^40^ Overcoming these barriers is crucial for enabling hydrogen's role in sustainable energy systems, particularly in the transportation and industrial sectors.

The present research provides a fundamental study on suitable material selection to fabricate state-of-the-art linerless full carbon fibre composite pressure vessels for hydrogen storage with the integration of MXene nanoflakes. Carbon fibre laminates were enhanced with MXene because of its superior strength, higher surface area, superior interfacial bonding, and higher aspect ratio, which collectively improve the load-transfer efficiency and hydrogen permeation of the pressure vessel. The tensile properties of the CFRP with UD and twill patterns were investigated, and the experimental results of tensile strength and modulus of CFRP provided a reasonable agreement with numerical simulations. NDT testing was conducted to detect manufacturing flaws, while SEM was conducted to observe the fractured surface morphology. Energy-dispersive X-ray spectroscopy was conducted to check the precise composition of the MXene powder. Thermogravimetric analysis (TGA) and differential scanning calorimetry (DSC) were conducted to determine the thermal behaviour and thermal stability of the laminates. Microhardness testing was conducted to evaluate the surface hardness and microstructural uniformity. Permeation tests were conducted in a pure hydrogen environment to fully capture the composites' response under cyclic pressurization. The aim of this research is to classify and empirically validate the most mechanically and functionally appropriate carbon fibre/epoxy composites for Type V COPV fabrication proposed for pressurized hydrogen storage through a comprehensive fundamental characterisation.

## 2 Materials and methods

### 2.1. Fabrication materials

The carbon fibre composite laminate studied in this research is an industrial product fabricated by utilizing a thermosetting epoxy laminating resin system composed of Bisphenol A, along with an amine-based curing agent, that has been developed further with a portion of 60 wt% unidirectional and twill carbon fibre fabrics. The unidirectional fabrics have a layup of [0]_4_, and the employed twill system consisted of high-strength [0,90]_3_ stitched carbon fibre fabric with a finished laminate thickness of 1 mm. The selected properties of the UD, twill carbon fabric, and epoxy resin are listed in Table 1.

**Table 1:** Specifications of carbon fibres with UD and twill pattern and epoxy resin

Specifications	CF-UD	CF-TW	Epoxy resin
Weave pattern	UD	2 × 2 twill	—
Resin content (wt%)	40	40	—
Yarn count	3 K	3 K	—
Thickness of one ply (mm)	0.22	0.28	—
Curing temperature (°C)	80 °C for 6 h	80 °C for 6 h	—
Tensile strength (MPa)	4830	3758	80
Tensile modulus (GPa)	234	231	—
Density (g cm^−3^)	1.80	1.80	1.13–1.17

UD carbon fibre fabric was supplied by Easy Composites Ltd, UK, for this study. Epoxy resin (EL2), along with slow-curing hardener (A30-catalyst), was used to impregnate the carbon fibres, which were also procured from Easy Composites Ltd, UK. The hardener utilized is A30, which has a long shelf life of 95–120 min and is particularly recommended for resin infusion and filament winding of COPVs. According to the manufacturer's recommended guidelines, an epoxy-to-hardener mixing ratio of 70 : 30 was used. Accordion-like Ti_3_C_2_ MXene was purchased from Laizhou Kai Kai Ceramic Materials Co., Ltd, with the product name Ti_3_C_2_T_*x*_. According to the manufacturer's data sheet, these nanoparticles have a mesh size of ≥200 with ≥98% purity. Dimethylformamide (DMF) was procured from Sigma-Aldrich (Sigma-Aldrich Company Ltd, Gillingham, UK) with 99.9% purity.

### 2.2. Fabrication of composite laminates

Three sets of carbon fibre laminates were fabricated, including the unidirectional standard (UD-S) and MXene-incorporated unidirectional laminate (UD-M), while the other set includes the twill standard (TW-S) and MXene-integrated twill laminate (TW-M). The MXene nanoparticle composition was 0.3 and 0.5 wt% in the UD-M and TW-M laminates, respectively. To facilitate a higher-level dispersion of MXene nanoparticles, they were first added to 100 mL of DMF solvent, followed by bath sonication for 60 minutes. After complete dispersion, a weighed amount of epoxy resin was subsequently added into the mixture by hand agitation, followed by 3 h of sonication with a 15 minute pause after 30 minutes of sonication. In the next step, the beaker containing the solution was transferred to a hot plate inside a vacuum hood. The complete evaporation of DMF took place at 155 °C while constantly heating for at least 6 h with continuous stirring. The solution was weighed after fixed time intervals to ensure complete DMF evaporation. The solution was allowed to cool to room temperature before subsequent fabrication steps.

**Fig. 1:**
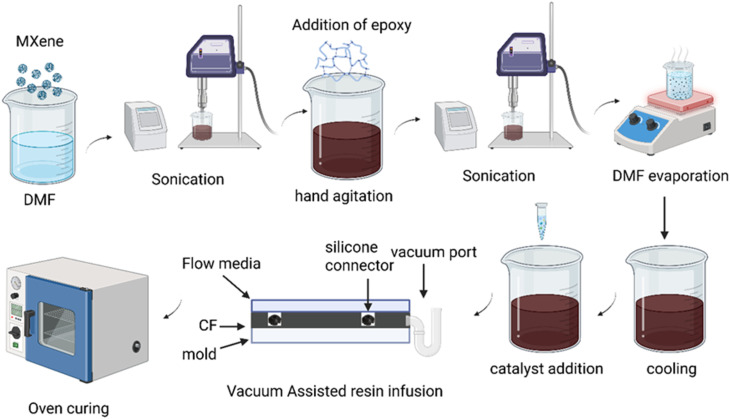
Schematic of the step-by-step procedure for fabrication of the composite laminate through sonication-assisted MXene dispersion and vacuum-assisted resin infusion.

The next stage of the sample preparation was the fabrication of the carbon fibre composite laminate following the vacuum-assisted resin infusion (VARI) process. A thick glass substrate was used as a base to create a resin infusion mould. The glass substrate was cleaned with acetone to remove any contamination from the surface. Then a mould-releasing agent was applied gently to the surface three times to ensure easy removal of the cured composite laminate. The carbon fibre fabric was cut into dimensions of 300 × 250 mm^2^, and plies were oriented in [0]_4_ for UD and three layers for the stitched [0,90]_3_ twill fabric. After carefully placing the carbon fabric layer by layer, the outer line of the mould was created with tacky tape. Resin infusion silicone connectors were then placed over the carbon fabric at opposing sides, as shown in Fig. 1, to allow inflow and outflow of the epoxy resin. Both connectors were connected using a stretched spring to allow good spread of the resin flow. Then a peel ply fabric was placed over the carbon fabric, which entirely covered all areas inside the mould. The use of peel ply here is mandatory to ensure easy separation of the laminate from top vacuum bagging. Then a flow media was applied over the peel ply, which covered the complete fabric area to facilitate the resin flow. Lastly, a vacuum bagging film was used to cover the mould. Both silicone connectors were then attached with a transparent tube, which acted as resin inlet and outlet pipes, and sealed. The outlet pipe was directly connected to the vacuum machine. To test the gas entrapment, the vacuum pump was turned on and switched off once the assembly was completely deformed. The resin inlet and outlet tubes were then clamped forcefully to ensure no air entrapment. After 10 minutes, it was examined to assess whether the assembly was still completely deformed as before; if not, then leakage should be carefully looked at and re-sealed.

For a given two-part epoxy system, the resin-to-catalyst weight ratio was fixed at 100 : 30. The mixing of the epoxy resin produced bubbles, which slowed down the flow of resin as well as reducing the mechanical integrity of the laminate. A degassing apparatus was used to eliminate the bubbles from the epoxy resin. Once mixed properly, the inlet pipe was placed inside the solution and clamped off. The already created vacuum helped suck the resin and started impregnating the fibres. The aim is to completely wet the carbon fibres and make certain that the resin is completely reinforced. Once all the resin flowed through all the parts of the mould, the resin inlet pipe was cut off, followed by the outlet pipe.

After complete impregnation, the excess resin was collected in a pot placed inside the vacuum pump. The pre-curing of the composite laminate took place at room temperature inside a fume hood, followed by post-curing at 80 °C for 6 h. For standard carbon epoxy laminates, epoxy was mixed with hardener, degassed, and impregnated the fibres under vacuum. Fig. 1 illustrates the fabrication mechanism used in this study.

The geometric dimensions of the samples for tensile testing were 250 mm × 15 mm × 1 mm with four plies for the UD laminate and 250 mm × 25 mm × 1 mm with three plies for the twill laminate. Aluminium end caps bonded to the tensile samples were prepared in two dimensions following the sample width (*e.g.*, 50 mm × 25 mm × 1 mm for twill and 50 mm × 15 mm × 1 mm for UD).

#### 2.2.1. Coupons fabrication for hydrogen permeation testing

Circular carbon fibre coupons with a diameter in the range of 85–90 mm were fabricated. A total of 9 UD samples were fabricated, including 3 virgin and 3 samples each at 0.3 wt% and 0.5 wt% loading. Each specimen had four UD carbon fibre layers, so that a 1 mm thick laminate is formed after complete curing. A hand layup method was used to impregnate all the carbon fibres using a soft brush. A thick glass substrate was used as a base, which was thoroughly cleaned with acetone to remove dirt and other contaminants. Three coats of mould-releasing agent were then gently applied on the surface, ensuring the evaporation of solvent before each successive coat. EL2 epoxy resin along with A30 slow curing hardener was mixed at 100 : 30, and complete homogenization was ensured prior to fabrication. A thin layer of epoxy was applied to the mould surface, and a single carbon fabric layer was placed over it. A bristle roller was used to remove excess resin from the base as well as any entrapped air bubbles. The top side of the fabric was then impregnated with resin, followed by impregnation of 4 fabric layers. For MXene-integrated epoxy CFRP composites, the same dispersion and evaporation strategies were adopted as discussed in Section 2.2, followed by a hand-laminating process and creation of the vacuum. Pre-curing of all samples took place under vacuum at room temperature inside a fume hood; however, post-curing took place in an oven at 80 °C for 6 h while the whole assembly was under high vacuum.

### 2.3. Finite element modelling for tensile testing

Finite element analysis (FEA) was conducted using ANSYS Workbench 2025R1 in conjunction with the ACP (pre) module to simulate the mechanical response. The laminate layup, material properties, and boundary conditions were defined in ACP (pre), while meshing was carried out in ANSYS Mechanical with an element size of 0.44 mm to ensure adequate resolution of the geometry and damage-prone regions. A mesh sensitivity analysis was conducted to evaluate the influence of mesh refinement on the simulation results, and convergence was achieved at approximately 19 210 elements and 19 810 nodes. Unidirectional carbon fibre laminates were stacked in four layers, and three layers were used for the twill pattern; both had a total thickness of 1 mm. The tensile component of the carbon fibre laminate was modelled using a 3D 8-node structural solid element (SOLID185) within ANSYS, implemented through the Composite PrePost (ACP) module. The laminate layup was defined with selected ply orientation and material properties; hourglass control was managed through element formulation settings to ensure numerical stability during reduced integration. During the tensile test, the boundary conditions were constrained in the upper and lower fixtures. The lower clamp of the test specimen was fixed in all directions, whereas the upper clamp was fixed in all directions but unconfined in the longitudinal direction. To accurately represent the boundary conditions during simulations, the upper and lower aluminium end tab positioning was defined as coupling constraints to define the interaction conditions. The reference point (RP-1) was created as the geometric centre of width and thickness. The degree of freedom was limited in all three direction displacements and rotations. Table 2 includes modelling data on the mechanical properties of the UD and twill standard and MXene-integrated carbon fibre composite laminates. The layer-to-layer delamination conditions in the composite laminate were simulated using the cohesive element model.^41–43^ For the traction separation approach, the constitutive model of cohesive elements provides an idealized representation of the complex fracture mechanism. The mixed-mode linear energy-based damage propagation criteria were used, and the Benzeggagh–Kenane (BK) law was implemented. The fracture energy *G*_c_ is given by eqn (1) and (2).1
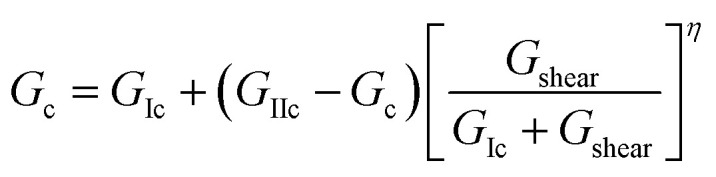

2 *G*_shear_ = *G*_Ic_ + *G*_IIIc_

where *G*_Ic_, *G*_IIc_, and *G*_IIIc_, respectively, represent mode I, mode II, and mode III fracture toughness, and *η* is the mixed mode parameter.

**Table 2:** Material's property dataset utilized in numerical modelling of the composite laminate

Properties	UD-S	UD-0.3 M	UD-0.5 M	Twill-S	Twill-0.3 M	Twill-0.5 M
Density (g cm^−3^)	1.62	1.62	1.62	1.5	1.505	1.507
Young's modulus in *X*-direction (GPa)	164.73	181.2	192.2	70.9	80.8	87.4
Young's modulus in *Y*-direction (GPa)	164.73	181.2	192.2	70.9	80.8	87.3
Young's modulus in *Z*-direction (GPa)	3.1	3.41	3.62	6.1	7.2	7.81
Poison ratio in *xy*	0.245	0.245	0.245	0.275	0.27	0.26
Poison ratio in *yz*	0.245	0.245	0.245	0.32	0.31	0.301
Poison ratio in *xz*	0.35	0.35	0.35	0.32	0.31	0.301
Shear modulus *XY* (GPa)	61.011	67.112	71.21	4.5	5.2	5.63
Shear modulus *YZ* (GPa)	61.011	67.112	71.21	2.3	2.8	3.11
Shear modulus *XZ* (GPa)	1.148	1.26	1.32	2.3	2.8	3.11

**Composite composition percentage**						
Carbon fibre (%)	60					
Resin (%)	40					

The effective displacement *δ*_m_ (eqn (3)) was introduced, which considers the damage caused by the combined effects of the normal and shear deformation. The damage variable *D*_c_ (eqn (4) and (5)) was calculated during the linear degradation stage and is defined as follows:3
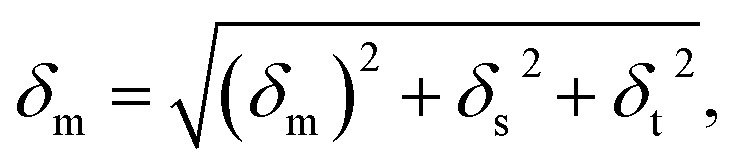
4
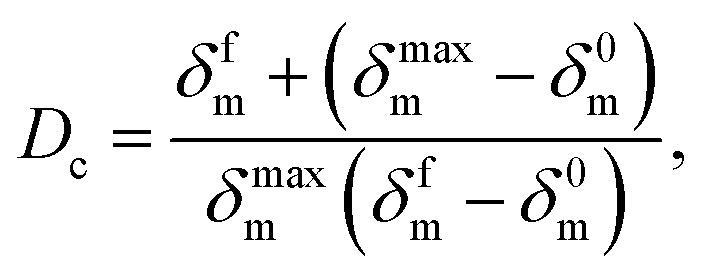
5
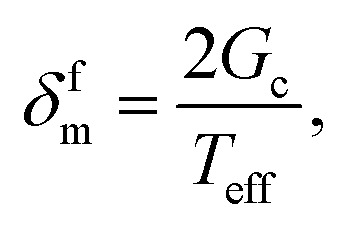
where *δ*^f^_m_ is the effective displacement at complete failure, with *T*_eff_ as the adequate traction at damage initiation. *δ*^max^_m_ refers to the maximum effective displacement achieved. Some data for the cohesive layers were acquired from the literature.^41–43^

In the developed FEA model, the effect of MXene as discontinuous reinforcement was integrated through engineering of the effective orthotropic material properties of the laminate. The MXene nanoflakes were not explicitly modelled geometrically at the nano-scale owing to the associated costs of multi-scale modelling approaches. As a substitute, the effect of MXene incorporation was demonstrated using homogenized material properties, such as Young's modulus, shear modulus, and stiffness-related orthotropic parameters. The modified properties relating to 0.3 wt% and 0.5 wt% MXene integration were allocated to the laminate plies in the finite element model to evaluate their effect on tensile properties and stress distribution. This homogenized approach has been extensively implemented for simulations of nano-reinforced laminated composites while preserving computational effectiveness and consistent prediction of structural response.

### 2.4. Mechanical characterization techniques

#### 2.4.1. Tensile testing

Tensile testing was conducted to study the tensile properties of the UD and twill carbon fibre laminates as well as MXene-integrated laminates. The tensile testing was performed on an Instron 3382 (Instron Corporation, Norwood, MA, USA), a universal testing machine with a maximum load capacity of 100 kN. The tensile tests were conducted on specimens with dimensions of 250 mm × 15 mm × 1 mm for UD and 250 mm × 25 mm × 1 mm for twill, following ASTM D3039, and the test speed was set at 238 mm min^−1^.^42^ For each type of laminate, a minimum of 5 samples were tensile tested.

#### 2.4.2. Microhardness testing

Vickers hardness testing was conducted with a microhardness tester HM-200 equipped with a diamond-shaped indenter, following ASTM E384. Before testing, the sample surface was cleaned and polished to obtain accurate measurements. Samples with dimensions of 100 mm × 50 mm were then carefully placed on the testing stage. Depending upon the material rigidity, a specific load of 2 N was applied to the surface through the indenter. The indenter approached the surface, and the actual dwell time was set at 10 seconds to ensure consistent indentation. After removing the load, the resulting square-shaped indentation was observed, and the lengths of the two diagonals were measured. The average of five tests was recorded per sample.

### 2.5. Microstructural characterisation

#### 2.5.1. SEM imaging parameters

Scanning electron microscopy (SEM) was conducted to examine the fractured surface of the carbon fibre laminates. The fractured samples were first cut into suitable sizes to fit perfectly into the SEM sample holder. The fractured surfaces were then cleaned using a soft brush to remove any loose debris without altering the surface features. To prevent charging during imaging, a thin conductive coating of gold was applied. The coated fractured samples were then mounted on aluminium stubs using conductive carbon tape to ensure proper electrical grounding. The images were taken under vacuum at a pressure of 100 Pa and a voltage of 25 kV. The fractured surfaces were scanned at various magnifications to observe fibre pull-out, matrix cracking, debonding, fibre breakage, brittleness, and plasticity, *etc.*

#### 2.5.2. Elemental mapping of MXene with EDXA

The energy-dispersive X-ray analysis (EDXA) was conducted in conjunction with scanning electron microscopy to determine the elemental composition of the MXene nanomaterials. A small amount of MXene powder was gently spread over the conductive carbon tape fixed on an aluminium SEM stub. The samples were coated with a thin conductive layer of gold to reduce charging during analysis. The fabricated samples were first analysed using SEM to locate a suitable area for analysis. The EDXA detector was operated at a voltage of 20 kV. The detected X-rays were processed to determine the elemental composition of MXene.

### 2.6. Non-destructive testing

#### 2.6.1. Ultrasound setup and measurement technique

Both UD and twill carbon fibre composite laminates were examined using Dolphitec Cam 2. This equipment has a 2D matrix array architecture, featuring a transducer module (TRM) that generates an impressive 16 384 elements distributed across an active aperture measuring 25 × 25 millimetres. This technically refined setup is executed *via* the precise orthogonal alignment of 128 transmitting electrodes, yielding elements with an exceptionally fine pitch of 0.25 millimetres at each intersection point. The equipment's outstanding data-gathering and resolution capacities are strengthened by its wide frequency spectrum, covering from 1.5 MHz to 10 MHz, thus aiding the inspection of an extensive range of material chemical compositions.

In the present research, we selected a 3.5 MHz transducer (labelled as TRM-EA-3.5 MHz) due to its deep penetration potential at medium frequencies. Process parameters were diligently chosen; gain was set at 6.5 decibels, and velocity for 1 mm thick carbon fibre laminate was set at 2777.78 m s^−1^. Once the data were captured, the regions of particular interest were identified and separated by key geometrical features (including circles, lines, and rectangles) by adopting advanced post-processing features. These innovative tools enabled the accurate evaluation of defects or delamination, including length, diameter, circumference, and depth, all of which are documented in millimetres under the strict criteria of the study protocol.

### 2.7. Hydrogen permeation testing

Hydrogen permeation testing through carbon fibre composite coupons integrated with MXene at different concentrations was conducted with manometric equipment featuring a closed chamber and varying pressure ranges. Permeation testing was conducted at low pressure due to equipment limitations and is intended as a comparative screening method rather than full-service validation. These tests were performed by strictly following ASTM D1434-82.^44^ A Mark 4 instrument purchased from Versaperm was used. Circular plate specimens with diameters in the range of 85–90 mm were utilized for permeation measurements. The composite specimen was placed in the sample holder directly connected to the upstream reservoir and to the vacuumed downstream reservoir, where the permeated hydrogen gas was collected. All the tests were conducted at a fixed temperature of 25 °C with 99.99% pure hydrogen gas. The system was not vacuumed overnight, but it was evacuated for at least 10 minutes before the start of the experiment to remove any entrapped gases from prior testing and to lower the background signal to accurately capture the hydrogen passed through the specimen. A 10 mbar pressure was maintained to start the test. A Druck gauge manometer was used to continuously monitor the upstream gas pressure, which was maintained at 5 bar throughout the experiment for all specimen; the downstream reservoir was kept under high vacuum to create a pressure gradient, which was the driving force for gas to diffuse through the sample. All specimens were exposed to hydrogen for an entire day. The experiment was stopped once the downstream pressure increased by 10 mbar. Due to its high sensitivity, a manometric measurement method was used to detect the pressure rise in the closed chamber. Hydrogen permeation was calculated using the following formula.6
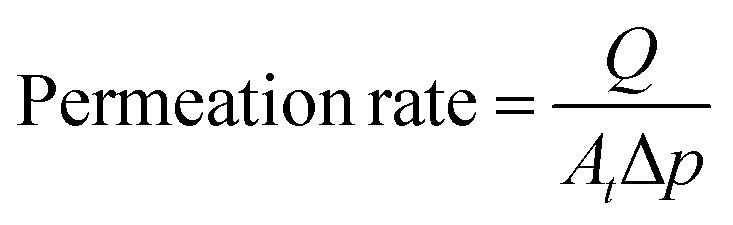


*Q* = volume of permeated hydrogen gas (mL), *A* = exposed area (m^2^), *t* = time (days), Δ*p* = pressure difference across the specimen (atm).

## 3 Results and discussion

### 3.1. EDXA analysis of MXene

**Fig. 2:**
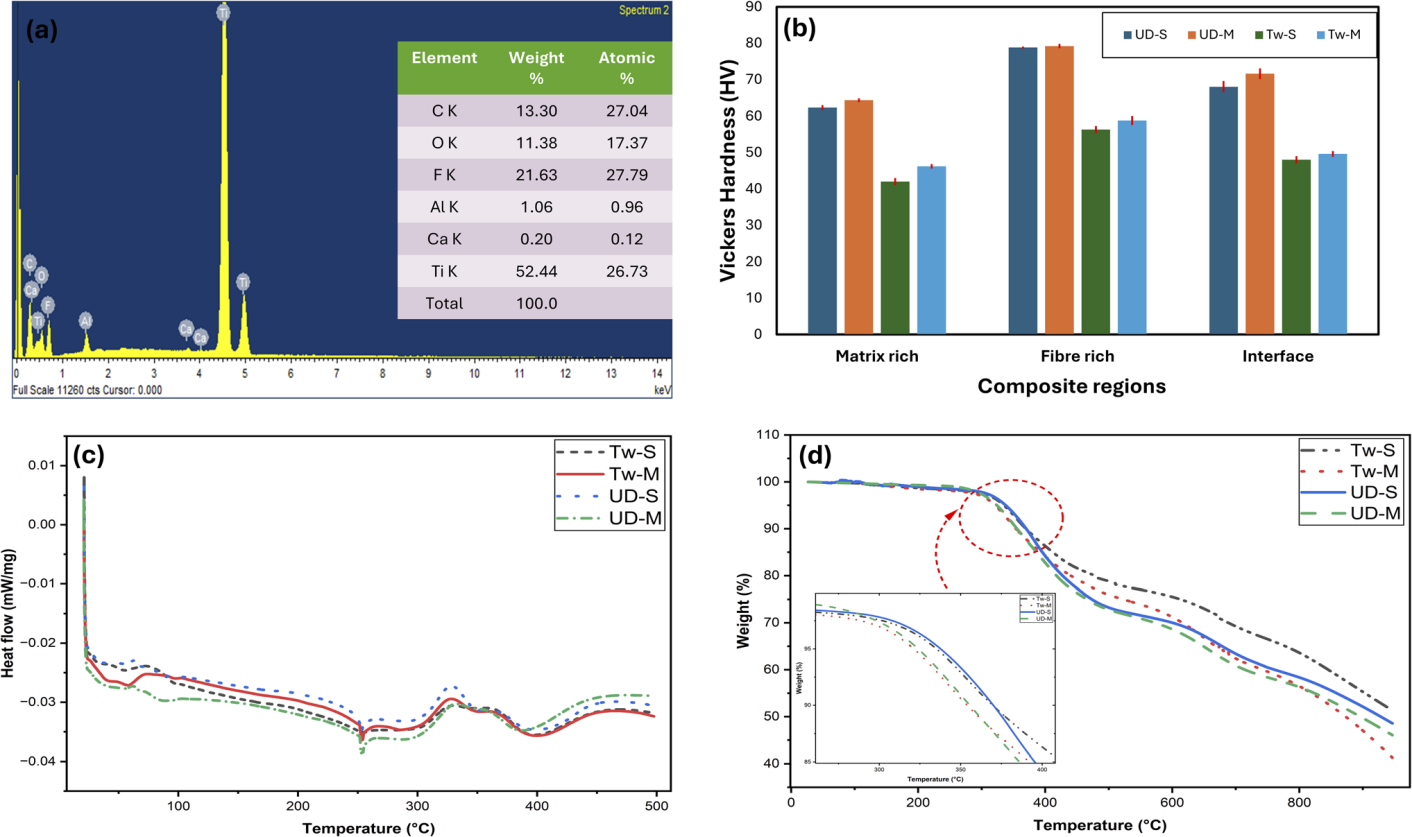
(a) EDXA spectrum displaying the elemental composition of MXene. (b) Vickers hardness testing at distinct composite regions. (c) DSC curves of the standard and MXene-integrated epoxy laminate composites, and (d) TGA curves of the standard and MXene-integrated epoxy laminate composites.

The elemental composition of as-received Ti_3_C_2_T_*x*_ MXene powder was investigated using energy-dispersive X-ray analysis (EDXA) to authenticate the effective etching of the MAX phase precursor (Ti_3_AlC_2_). The spectrum obtained through EDXA (Fig. 2(a)) identified that titanium (Ti), carbon (C), and oxygen (O) are predominant elements. The presence of oxygen could be attributed to the surface oxidation during etching or air exposure. Furthermore, residual traces of fluorine, aluminium and calcium were found, likely inherited from their MAX phase precursor (Ti_3_AlC_2_). Detected weight percentages of titanium and carbon at 52.44 wt% and 13.30 wt%, respectively, support the formation of a Ti_3_C_2_ phase. Notable oxygen traces of 11.38 wt% were recorded, potentially due to the presence of surface terminations of –O or exposure-related oxidation during handling. Detected traces of aluminium and fluorine with 1.06 wt% and 21.63 wt%, respectively, are attributed to residuals from both the MAX phase and fluoride-based acid etching. The observed elemental composition aligns well with known Ti_3_C_2_ MXene characteristics, resulting in the successful synthesis of a 2D structure with –O, –OH, and –F as surface terminations (T_*x*_). These terminal groups play a pivotal role in enhancing MXene dispersion efficiency and chemical reactivity.

### 3.2. Microhardness testing

Six indentations were made on each sample, and the resulting HV values were plotted against matrix-rich zones, fibre zones, and interfaces (Fig. 2(b)). The indentation results revealed that unidirectional (UD) CFRP laminates exhibited higher Vickers hardness (HV) values in all regions compared to twill laminates. This consistent improvement in the UD laminate is due to the perfect fibre orientation, which offers optimum load transfer efficiency and localized stiffness in the indentation direction. In one study, the researchers conducted Vickers microhardness testing on a unidirectional carbon fibre reinforced polymer (CFRP) laminate with a thickness of 1.2 mm. The reported hardness values ranged from 88.45 to 100.28 HV, demonstrating high hardness values that reflect outstanding stiffness.^45^ The Vickers hardness value calculated in the current research for the unidirectional CFRP laminate is 80 HV, which is a little lower compared to values reported in the literature due to the presence of manufacturing defects in the form of voids or dry fibres. UD laminates, due to their aligned fibres, demonstrate enhanced hardness stability under the Vickers indenter; however, the twill laminates, influenced by interlacing points, fibre crimps, and higher resin-rich regions, lead to lower and more inconsistent hardness. The inherent crimp and interlacing of the twill laminates foster stress concentration zones and often initiate matrix cracking during indentation.

Notably, the MXene-integrated laminates at low concentrations of 0.3 wt% – both UD-M (79.2 HV) and twill-M (58.8 HV) – resulted in improved hardness values as compared to their standard laminates, with 78.8 HV and 56.3 HV, respectively. However, the MXene-integrated unidirectional laminate (UD-M) exhibited slightly higher and more consistent hardness values across the identified regions. To begin with, the presence of MXene strengthens the load transfer between epoxy and carbon fibres through their crack-bridging and arresting features and mitigates microcrack initiation and propagation under the indenter. MXene also promoted microstructural refinement within the epoxy matrix, resulting in a stiffer and tougher resin phase. The integration of MXene created an internal reinforcement channel that limits plastic flow and crack initiation under localized stress. MXene supported better resin flow and nucleation, resulting in lower void content, enhanced fibre wet-out, and more uniform curing of the laminate. The MXene-integrated UD laminates imparted directional stiffness due to fibre alignment, simultaneously strengthened the fibre-matrix interface, and improved crack resistance. Similarly, the MXene-integrated twill laminate demonstrated a slightly higher HV value compared to that of the standard twill variant, showcasing the combined impact of fibre orientations and MXene enhancement. It has been reported that Vickers hardness testing across various regions of the bulk-fill polymeric resin composite reveals a distinction between fibre-rich, resin-rich, and interfacial regions. The results indicate that the fibre-rich region (laminate top surface) displayed the highest hardness value of 61.5 HV, while the resin-rich region (bottom surface) displayed a lower hardness value of 47.8 HV, and the interface region (lateral surface) revealed a moderate hardness value of 54.2 HV. The observed differences stem from disparities in polymerisation and compositional heterogeneity through the thickness of the laminate.^46^

Taken together, these findings demonstrate the importance of fibre alignment, matrix integrity, and nanofiller integration to affirm the laminate's surface mechanical response. The presence of MXene strengthens the laminate at the microscale, offering the potential to improve mechanical endurance and structural stability during prolonged use. The UD-M laminates offer the highest specific strength-to-weight ratio and precise fibre alignment with minimal manufacturing defects, making them ideal for the fabrication of high-performance, lightweight Type V COPVs.

### 3.3. Differential scanning calorimetry

Differential scanning calorimetry (DSC) was conducted on 4 types of carbon fibre laminates to evaluate their thermal transition, degree of cure, and glass transition (*T*_g_) behaviour (Fig. 2(c)). All the laminates revealed clear and consistent DSC thermograms, reflecting similar trends across all types of laminates. The glass transition temperature, a clear baseline shift in the DSC curves around 75 °C, corresponds to the increased molecular motion in the epoxy matrix. The detection of a translucent glass transition in all composite laminates indicates complete cross-linking and uniform curing. Subtle shifts in *T*_g_ among all the carbon fibre composite laminates could stem from fibre orientation, resin distribution, fabrication procedure, and post-curing conditions. However, unidirectional laminates possess denser fibre content with limited resin flow channels, which may restrict the chains' mobility and result in slightly increased glass transition temperatures.

The laminates enhanced with MXene displayed improved thermal performance. The DSC curves of the MXene-integrated samples display an upward shift in the *T*_g_ and a reduced transition region, indicating improved cross-linking density and restricted molecular movement. This enhancement in the *T*_g_ is attributed to the presence of MXene as nano-reinforcements, which created strong chemical connections with the epoxy resin. This strong chemical bonding limits polymeric molecular chain movement, fostering a more rigid thermoset matrix.

The integration of MXene serves a dual role as better thermal control and a nucleation centre during processing, aiding in achieving more uniform curing with a lower void content. Owing to MXene's larger surface area and elongated geometry, it facilitates a percolating network that impedes molecular motion, effectively enhancing the *T*_g_ and thermal resilience. MXene-integrated carbon fibre composite laminates display superior thermal properties, achieved through matrix rigidity, improved interfacial bonding, and a uniform curing process. The observed improvement in the *T*_g_ suggests improved suitability for high-performance composite applications requiring thermal endurance and sustained structural integrity, particularly under prolonged thermal stress, where long-term dimensional and mechanical stability is critical.

### 3.4. Thermogravimetric analysis

Thermogravimetric analysis (TGA) was conducted on carbon-fibre-reinforced polymer (CFRP) composites, composed of both standard and MXene-integrated systems, to study their thermal stability and decomposition behaviour. All four samples displayed clearly defined thermograms with similar weight degradation trends, indicating homogeneity in the matrix composition and consistent arrangements of reinforcement. All the TGA thermograms (Fig. 2(d)) exhibited two-stage weight loss. Initially, a minor weight loss was evident below approximately 375 °C, linked to the evaporation of loosely bound moisture and small volatile fractions. A more significant weight loss occurred in the second stage, between 375 °C and 450 °C, representing the thermal decomposition of the epoxy resin. Above this temperature, the remaining mass is mainly comprised of carbon fibre and heat-resistant additives. Across the unmodified samples, only slight variations in the degradation were observed, suggesting that fibre alignment has a minimal effect on the thermal resilience of the epoxy resin system. The integration of nanomaterials led to improved thermal performance and diminished weight loss during decomposition. This enhancement is associated with the barrier effect and thermal shielding of the MXene, which restrains the diffusion of decomposed gases and prolongs the decomposition time. The formation of char yield identified at elevated temperatures in MXene-integrated samples further strengthens this interpretation, as the integration of MXene contributes to the generation of more heat-resistant carbon residue.

In summary, the TGA findings indicate that all the samples demonstrate consistent thermal stability, affirming their suitability in high-end structural applications. The addition of MXene contributes to improved fire resistance, thermal endurance, and long-term durability.

### 3.5. Non-destructive evaluation (NDE)

#### 3.5.1. Twill CFRP (Tw-S) laminate

**Fig. 3:**
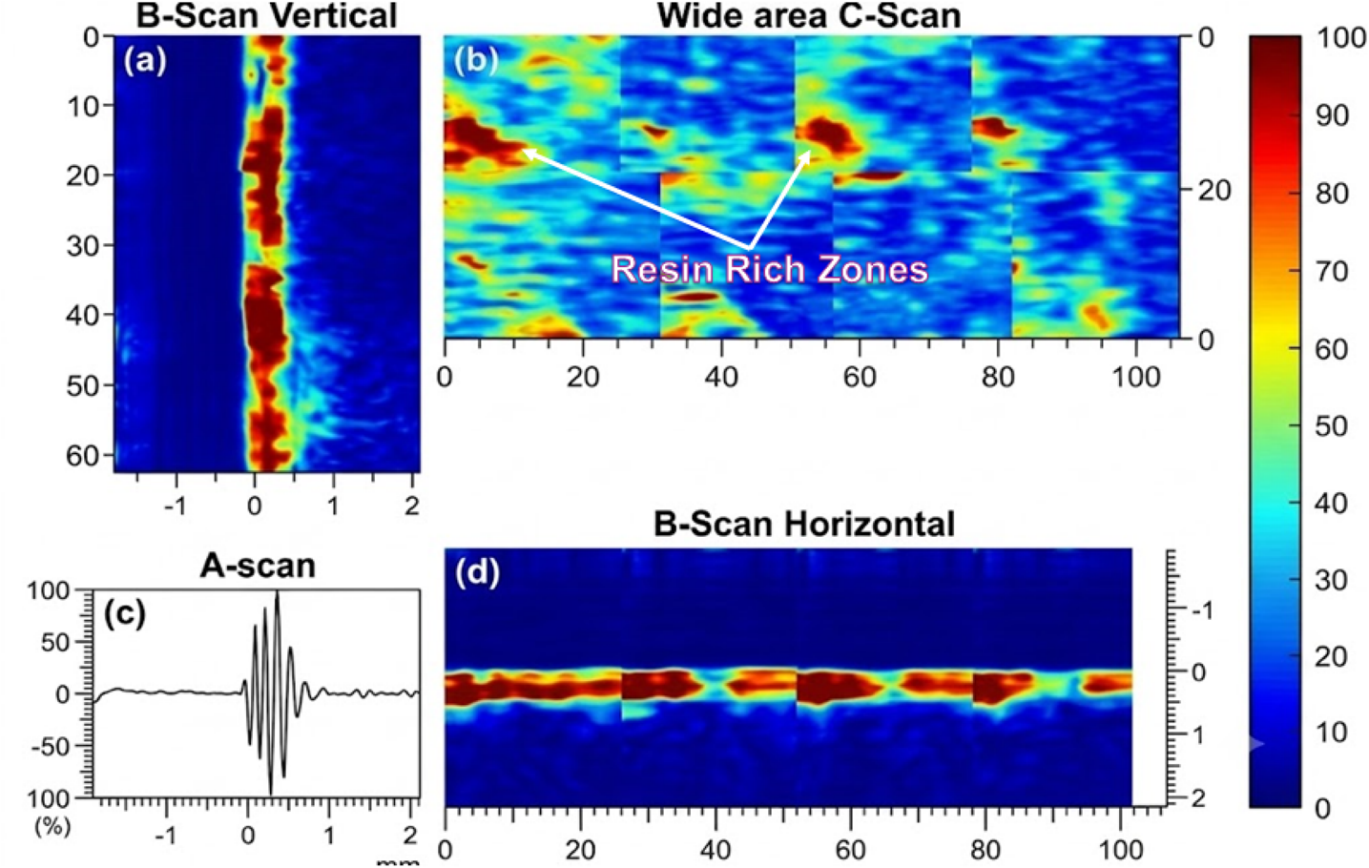
The A-, B- and C-scans of the Tw-S laminate, with (a) the B-scan in the vertical direction, (b) the C-scan stitching, (c) the A-scan in the vertical direction, and (d) the B-scan in the horizontal direction.

The ultrasound analysis of 1 mm thick twill CFRP laminate was conducted to reveal different manufacturing defects across A-, B-, and C-scan stitching (Fig. 3). The A-scan (image c) exhibits an irregular intermediate amplitude response, including several low-intensity echoes following the primary back wall interface. These findings suggest the presence of micro-voids, inclusions, or acoustic impedance mismatch, likely introduced by resin-dominated regions. The B-scan (image d) provided details on the through-thickness signal disruptions and attenuated amplitudes in the exhibited zones, indicative of poor resin distribution. The broken signal provides evidence of localised areas with dry fibres or uneven vacuuming during curing. The C-scans (image b) displayed a mix of high- and low-amplitude reflection zones distributed non-uniformly across the scanned surface. The observed amplitude response may be attributed to air entrapment or resin deficiency. The analysis revealed a remarkably high-amplitude zone quantified at 660.52 mm^2^, pointing towards the defect-dense zone. This area represents less consolidation and higher resin viscosity. Highly defective areas are shown in red colour, stemming from vacuum bagging efficiency or incomplete resin infusion. Collectively, the scan analysis reveals the presence of internal defects in the form of voids, air entrapment, and inhomogeneous resin distribution across the laminate's internal structure.

#### 3.5.2. Twill CFRP laminate (Tw-M) integrated with MXene

**Fig. 4:**
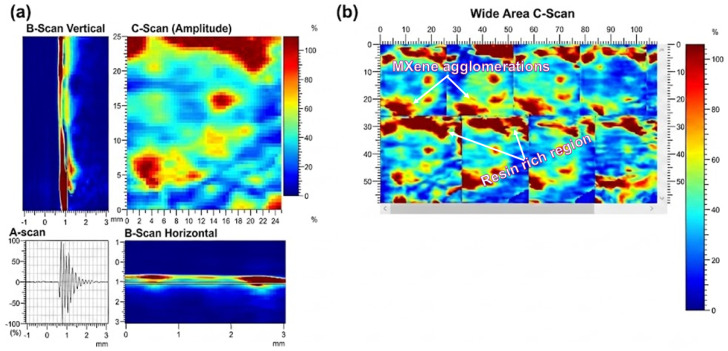
Images of (a) the B-scan in the vertical direction, the C-scan amplitude, the A-scan in the vertical direction, the B-scan in the horizontal direction, and (b) the C-scan stitching of the Tw-M laminate.

The A-scan data revealed high-amplitude reflections, reflecting substantial acoustic impedance mismatch within the MXene-integrated twill CFRP laminate (Fig. 4). The peak amplitude exceeded 80% of the total height and was attributed to significant MXene agglomerations, while multiple low-intensity signals suggest interlayer manufacturing defects through a thickness of 1 mm. The C-scan amplitude in image (a) highlights spatial heterogeneity, with higher amplitude zones in the upper right quadrant suggesting optimum fibre/resin adhesion and notably attenuated regions near the left lower end representing manufacturing-induced defects such as porosity or MXene agglomerations. Within the MXene-incorporated laminates, these attenuations represent localized nanoflake agglomerations, which affect the fibre matrix continuity and initiate the acoustic scattering heterogeneities. B-scans also affirm that the scattering in the mid-ply laminate is consistent with through-thickness epoxy-rich regions; however, the A-scan highlights secondary reflections, which are collectively characteristic of twill fibre architecture and inhomogeneous mixing of MXene nanoflakes within epoxy resin.

The stitching of C-scans in image (b) displays high-intensity regions within the central region, representing good consolidation of a composite laminate having uniform material properties. The scattered dark zones are suggestive of strongly reflective features, like dry fibres or MXene agglomeration in resin-rich areas. The yellow arrows also indicate transitions from high- to lower-amplitude zones, which correspond to fibre waviness, a defect commonly induced by residual stresses during curing in the twill laminates.

#### 3.5.3. UD CFRP laminate (UD-S)

**Fig. 5:**
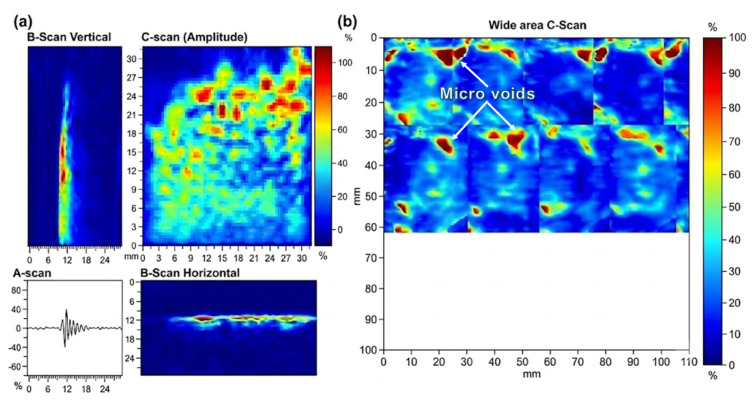
Images showing (a) the B-scan in vertical direction, the C-scan amplitude, the A-scan in the vertical direction, and the B-scan in the horizontal direction, and (b) C-scan stitching of the UD-S laminate.

This high-temperature post UD-S CFRP laminate was inspected to identify the internal manufacturing defects, such as voids, dry fibres, and resin-rich zones. A combination of A-, B-, and C-scans and mapping was used to locate and characterize the microstructural aberrations (Fig. 5). The vertical and horizontal B-scans, shown in image (a), highlight discrete high-amplitude reflection zones as bright yellow and red areas. These bright reflections represent interfaces where acoustic impedance alters immediately, pointing to possible micro-voids. The B-scan revealed a high-amplitude reflection zone from the middle to the bottom, representing subsurface irregularities, potentially aligning with the characteristics of epoxy pockets. Two localized reflections in the middle zone display near-surface voids or resin entrapment, causing impedance mismatch.

The A-scan waveform, shown in image (a), displays successive echo signals, the initial front surface response, and subsequent back wall reflections. The higher-amplitude peaks represent secondary peaks, which signify the interfaces inside the composite laminate, validating the detection of internal defects, such as voids, porosity or fibre misalignment. Time of flight echo represents the depth, displaying amplitude irregularities in the B-scan.

The C-scan, also showing the stitched mapping in image (b), displays localised regions of intensified signals scattered across the whole scanned area of the laminate. The red annotated hotspots observed in the original scan point to the presence of internal inclusions like entrapped air or resin-rich areas. The observed hotspot concentration panel points towards inconsistencies in resin flow during infusion or compaction during fabrication of the laminate. The high-intensity signals that appear in the C-scan represent air pockets. These defects are attributed to incomplete compaction during vacuuming, incomplete wetting of fibres, and thermal degassing.

#### 3.5.4. UD CFRP laminate (UD-M) integrated with MXene

**Fig. 6:**
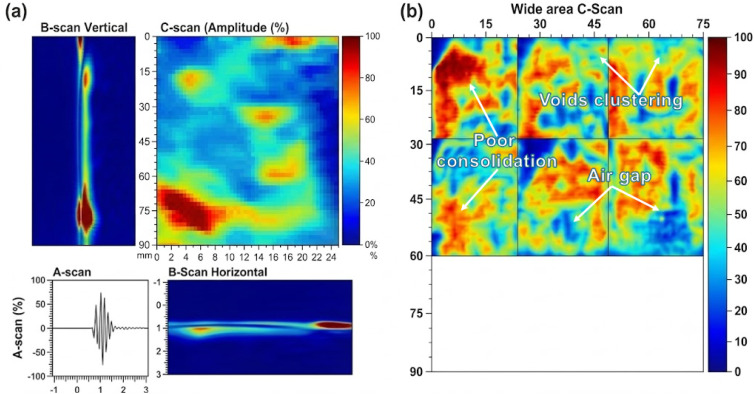
Images showing (a) the B-scan in the vertical direction, the C-scan amplitude, the A-scan in the vertical direction, the B-scan in the horizontal direction, and (b) C-scan stitching of the UD-M laminate.

Ultrasonication was employed to detect the internal manufacturing flaws in the unidirectional CFRP laminate enhanced with MXene (Fig. 6). The A-scan (image a) displays a specific series of reflections representing the front and back walls of the composite laminate. The observed signal fluctuations, coupled with successive smaller echoes, reflect the material inconsistencies. These are associated with micro-voids, poor impregnation or resin-rich areas inside the composite laminate. The amplitude attenuation at certain depths might reflect the presence of embedded defects or poorly wetted zones due to higher resin viscosity. Both horizontal and vertical B-scan (image a) images of the composite laminate revealed internal details spanning the laminate thickness. The scan displayed strong front and back wall signals at approximately 0.5 mm and 1.5 mm depth, representing the laminate thickness.

Weak ultrasonic signals can be observed between both surfaces, suggesting the presence of void clustering or internal imperfections. The horizontal B-scan highlights variable signal intensity, indicating planar defects, like matrix cracking and MXene agglomeration, that result in site-specific deviations in impedance response.

The C-scan (image b) mapping was conducted to capture the internal defect distribution across the planar areas of the laminate. High-intensity zones, identified by red and yellow, are intensely focused on the lower left region of the scanned area. These regions are often linked to structural imperfections like porosity or matrix depletion. The sky-blue medium reflections correspond to partial infusion or MXene agglomeration. The observed variation in the scanned profile suggests inconsistencies during processing, due to uneven vacuum bagging or incomplete resin infusion, or during MXene mixing. The stitched C-scans displayed wider trends across the specimen surface. The imaging data exhibit irregular defect occurrence within the laminate. All the stitched zones display matrix-cracking, resin-rich zones as well as air gaps (shown in blue). These inconsistencies are related to manufacturing-induced defects, including the thermal gradient during curing or resin/MXene concentration.

### 3.6. Tensile testing

**Fig. 7:**
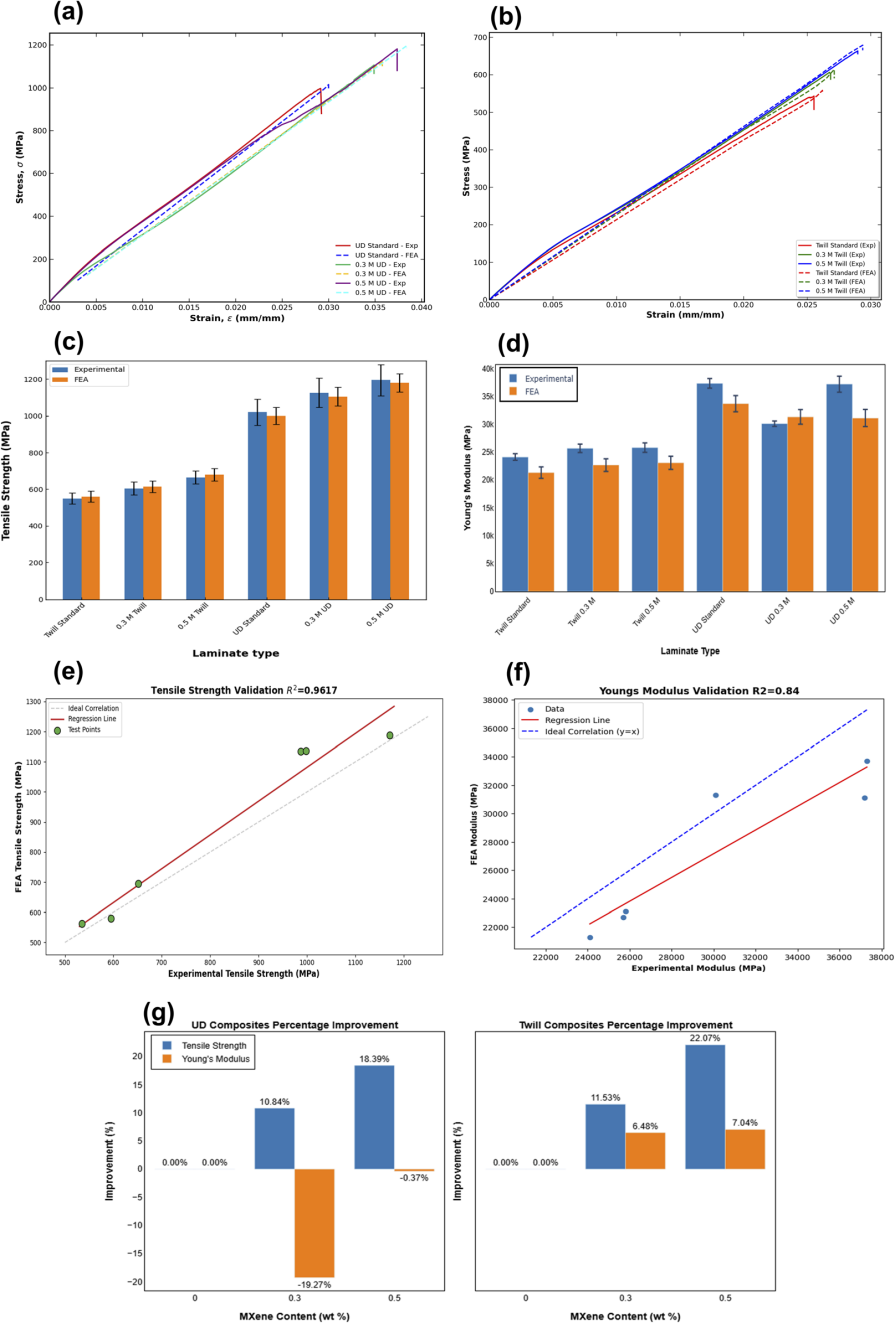
Influence of MXene incorporation and fibre orientation on mechanical properties and FEA model validation: (a and b) stress–strain plots demonstrating strength and modulus differences between UD and twill laminates; (c and d) quantitative evaluation of tensile strength and modulus improvements; (e and f) regression-based validation of FEA predictions against experimental data; and (g) percentage improvement in mechanical characteristics as a function of MXene concentration.

Fig. 7 highlights the tensile properties of the laminates. Image (a) shows the tensile stress–strain response of the UD and MXene-integrated CFRP laminates after experimental and FEA modelling. All the curves display a linear elastic response up to the failure point, which confirms the brittle nature of the composite for various laminate configurations. The UD-S laminate shows a tensile strength of 997.32 MPa, which rises to 1105.44 MPa at 0.3 wt% of MXene loading, and finally increases to 1180.76 MPa at 0.5 wt% of the MXene-integrated laminate, corresponding to an improvement of 10.84% and 18.39%, respectively. This improvement in strength is indicated by the steeper slopes for the MXene-integrated laminates, suggesting better load-transfer efficiency at the fibre matrix interface due to optimum adhesion. MXene-integrated UD laminates display lower Young's modulus due to manufacturing-induced defects relative to the standard UD laminate (43 147.37 MPa). The FEA simulation results are concurrent with the experimental findings; the tensile strength deviation trends are within 5–10%, which demonstrates a good agreement in capturing the fibre-governed tensile response; however, its predictive competence is constrained due to the non-appearance of defect and damage growth mechanisms. The marginal drop in stiffness near the failure point in FEA can be ascribed to idealized assumptions, including optimum interfacial adhesion, perfect fibre placement, no manufacturing-induced defects and damage progression.

Image (b) displays the stress–strain response of experimental and FEA studies on the standard and MXene-integrated twill CFRP laminates. All the curves exhibit linear elastic behaviour up to the failure point due to their inherently brittle nature. The standard twill laminate has a tensile strength of 543.6 MPa, increasing to 606.3 MPa at 0.3 wt% MXene loading and finally to 663.5 MPa at 0.5 wt% of the MXene-integrated laminates, demonstrating an improvement of 11.53% and 22.07%, respectively. A consistent improvement in tensile strength is further supported by an enhancement in the tensile modulus, which increases from 26 779.82 MPa for the standard laminate to 27 955.41 MPa at 0.3 wt% and 28 398.08 MPa at 0.5 wt% MXene loading, which further validates the effectiveness of load transfer at the fibre-resin interface due to improved adhesion. The FEA simulations demonstrate a good agreement with the experimental findings for all the laminate systems, with a deviation in the maximum stress remaining below 10%, thereby validating the sturdiness of the materials modelling and assumed boundary conditions. All the presented FEA curves show an idealized linear elastic behaviour, which could be attributed to the assumption of homogeneous laminates' response and defect-free fabrication. Minor discrepancies are observed in the laminates integrated with 0.5 wt% of MXene near failure, where the experimental curve displays earlier failure signs relative to the simulation curve. This variation is linked to contained damage accumulation mechanisms, such as matrix cracking, fibre breakage, and debonding, which are not entirely described in the continuum-based modelling approach. A close connection between experimental and FEA simulation results, combined with consistent improvement in the structural response, validates the predictive capability of the developed FEA model and displays the usefulness of matrix modification in improving the tensile behaviour of the twill CFRP systems. Even though NDE discovered considerable localized delamination in the MXene-incorporated twill composites, the manufacturing defects were not homogeneously dispersed and thus did not entirely govern the overall tensile performance. The monitored increment in tensile strength indicates that MXene improves interfacial adhesion and load transfer in structurally intact zones. Yet, the findings also emphasize that the mechanical properties of the MXene-integrated composites are extremely vulnerable to processing quality, and manufacturing-induced defects may moderately conceal the true material capability.

Image (c) provides a comparative analysis of tensile strength, which displays a consistent rise for both UD and twill laminates enhanced with MXene, while the UD configurations highlight superior strength across all the laminate types. This rising trend in UD laminates is ascribed to the highly aligned fibres in the load direction, which promotes an effective axial load transfer and diminishes the stress concentration points; however, the crimped fibre architecture present in twill CFRP composites induces localized fibre waviness, which lessens the structural strength. The perceived enhancement with increasing MXene concentration indicates enhanced fibre/matrix interfacial adhesion, leading to better load transfer and delayed damage initiation and progression. A close match between FEA and experimental findings across all laminate types validates that the developed model well records the leading deformation mechanisms; however, minor variations originate from microstructural inhomogeneities that are not included in the simulations. From an application point of view, the consistently higher tensile strength shown by the UD composite systems makes them ideal for the fabrication of Type V COPV, where fibres are aligned in the circumferential direction to withstand the tensile stresses, and the hoop winding endures most of the hoop stresses generated during hydrogen pressurization.

A comparative analysis of Young's modulus is presented in image (d) where a consistent rising trend is not followed across configurations owing to their dependence upon stress transfer efficiency at the fibre/matrix interface. Both UD and twill laminates are sensitive to slight changes in fibre orientation and manufacturing-induced defects, such as void content, resin accumulations and composite consolidation, that do not necessarily scale with laminate configuration, as also observed for tensile strength. UD laminate configurations display higher modulus relative to twill laminates due to the aligned fibres and the absence of tow crimps, which makes the UD configuration modulus data a reliable input for Type V COPV fabrication, where accurate modulus prediction directly governs the wall thickness of the composite tank and the mechanical strength under cyclic pressure loading. However, increment in the layer number has minimal effect on the laminate modulus as stiffness is fibre orientation-dependent as opposed to thickness. The structural performance of MXene-incorporated composites is dominated by hostile processes, such as nanoflake dispersion, interfacial bonding, and the formation of defects. Although agglomeration can lower the modulus in the vicinity of MXene by disordering load transfer, the concurrent development of crack-bridging and interfacial bonding leads to enhanced tensile strength. These characteristics support the non-monotonic stiffness response together with a steady increase in strength.

A good correlation between tensile strength obtained from experiment and FEA simulations is displayed in image (e), where *R*^2^ = 0.9617, which supports how effectively the developed model captures the actual failure of the composites. Over the entire range of tested laminates, from the consistently low strength twill configurations to high integrity UD laminates approaching approximately 1200 MPa, the tight clustering of data points near the regression line provides a strong justification that material property inputs, boundary conditions and defined damage criteria were appropriate and well representative of the experimental tests. This level of agreement is noteworthy because it holds over various composite configurations, establishing that the developed model is not set for a certain fibre architecture, but it has sufficient competence to predict failure across various orientations.

The modulus validation displayed in image (f) provides an *R*^2^ of 0.84, which still represents a fair and physically meaningful level of agreement; however, the modest reduction in correlation relative to strength regression is not surprising from a mechanistic perspective. Nevertheless, the modulus is strongly influenced by microstructural features in comparison to tensile strength, and the appearance of small manufacturing defects can introduce scatter that a homogenized model cannot completely resolve. The capability of the developed FEA model to remain slightly lower than the ideal line at higher stiffness values indicates that it makes slightly conservative modulus predictions, which is favourable for simulating the actual pressure vessel since it avoids overestimating the composite's rigidity.

The percentage improvement plots for both laminate configurations, as displayed in image (g), highlight that the incorporation of MXene nanoflakes influences the tensile strength and stiffness quite differently; both behave differently as the nano-filler content increases, which itself is a technically noteworthy observation worth discussing. Both UD configurations display a consistent improvement in strength from 10.84% to 18.39% at 0.3 and 0.5 wt% loading, respectively, which reveals how well the dispersed MXene nanoflakes influence the load dissipation and arrest the advancing crack within the polymeric matrix. However, modulus expresses a different story: at 0.3 wt% of filler loading, it drops to −19.27% and rises again to −0.37% at 0.5 wt% loading. This significant drop in stiffness at 0.3 wt% MXene loading is ascribed to poor nanoflake dispersion and uneven load-transfer efficiency, which has the capability to disrupt the linear elastic response and hence reduce the modulus. In contrast, the tensile strength is influenced by damage initiation and propagation mechanisms like crack arrest and bridging, deflection, and optimum fibre-resin adhesion promoted by MXene's terminal groups. At 0.5 wt% MXene loading, the fillers significantly improved the reinforcement efficiency and stress distribution channels, moderately balancing the severe effects of confined nanoflake clusters, hence contributing to the partial recovery in stiffness while sustaining improved tensile strength. The experimental dissociation between tensile strength and modulus at lower nanofiller loading could be ascribed to the differential sensitivity of these characteristics to their clustering. At lower filler content, the agglomerates decrease the interfacial adhesion with the resin, hence compromising the modulus contribution of the filler and lowering the stiffness.^47^ However, the loose clustering of nanoparticles acts as a physical hindrance to advancing cracks by bridging and deflection, increasing energy absorption at the crack tip and peak load without inevitably contributing to modulus. Crack bridging has been identified as a toughening mechanism in multifunctional nanocomposites; such agglomeration may reduce the modulus due to a reduction in interfacial connections and diminished stress transfer efficiency, while crack-based energy dissipation remains active.^48^ Higher nanofiller loadings into polymeric matrices improve the spatial coverage and promote network formation, which effectively transfers the applied load, leading to a recovery in Young's modulus, which is coherent with the supremacy of the dispersed volume fraction over the clustered volume fraction in determining the stiffness.^49^ The twill configurations follow a consistent rising trend where tensile strength improves from 11.53% to 22.07% with a marginal improvement in stiffness from 6.48% to 7.04% at 0.3 and 0.5 wt% filler loading, respectively. These findings reveal that woven laminates manage higher MXene loadings better than UD configurations in preserving the modulus but still cannot entirely overcome the agglomeration issues. Collectively, the results confirm that MXene nanoflakes are highly effective nanofillers that offer substantial improvement in tensile strength; however, the fabrication methodology should be improved to overcome the challenges related to flake agglomeration owing to strong Van der Waals interactions. The increase in the tensile response observed in MXene-integrated composites is attributed to the enhanced effective orthotropic properties allocated within the homogenized laminate model.

The present research offers a fundamental assessment of MXene-incorporated carbon fibre laminates with a thickness of 1 mm, with their mechanical properties acting as reference materialinputs for actual Type V tank fabrication. The calculated tensile strength of 1180.7 MPa for the optimized 0.5 wt% MXene composite coupon shows improved load-carrying capacity at the material stage. In the framework of thin-walled pressure vessel theory, the experimental findings offer a preliminary screening tool for evaluating likely pressure endurance; however, real Type V COPV performance is controlled by supplementary structural aspects such as fibre construction, winding configuration, wall thickness (typically 10–14 mm in full vessels), safety aspects, and fatigue performance under cyclic pressurized events. So, the presented outcomes herein should be taken as material-level feasibility guides rather than full-scale structural performance validation, offering important insights for future optimization and scale-up toward 350–700 bar Type V COPV.

**Fig. 8:**
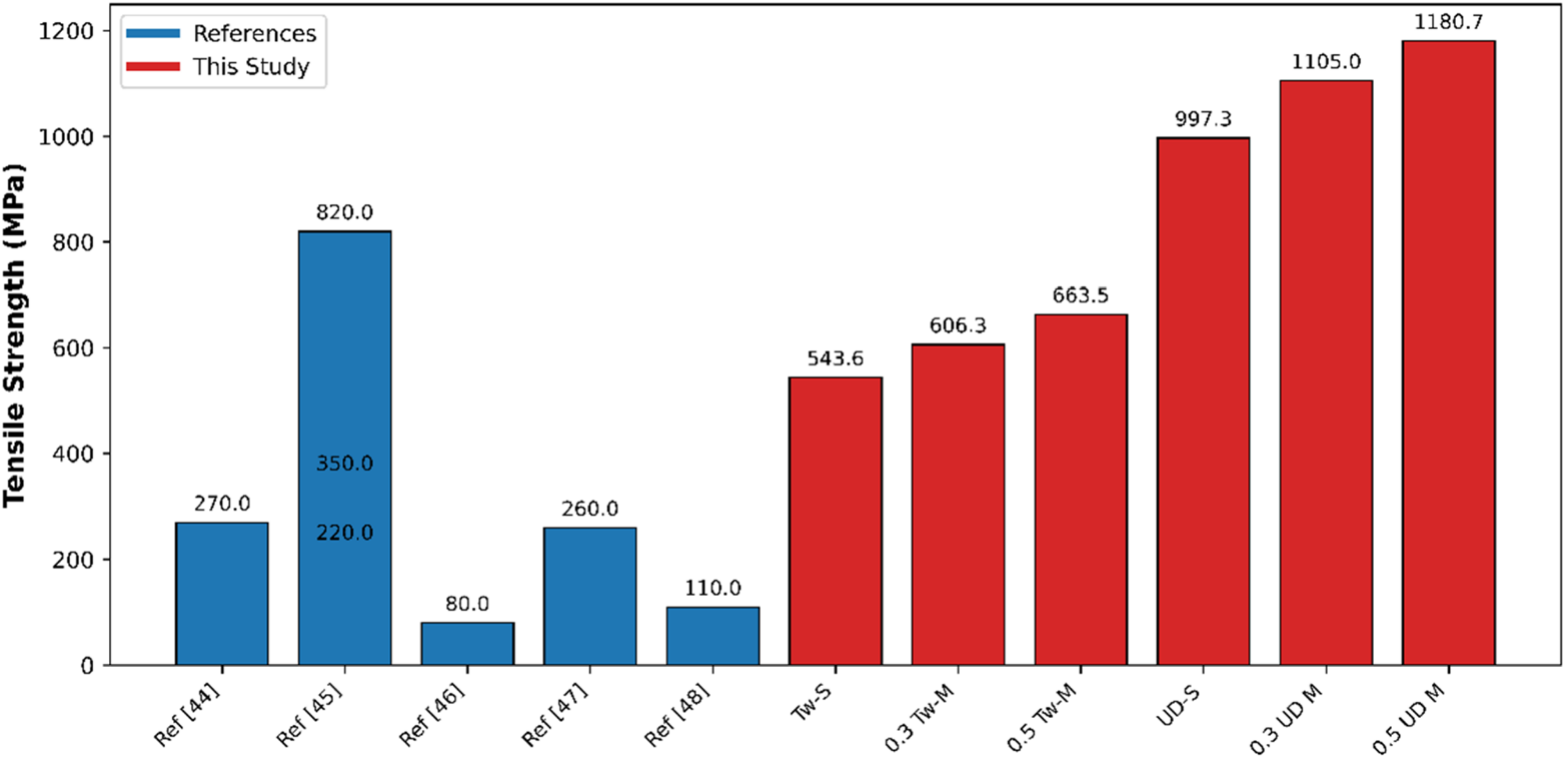
Comparative tensile strength analysis of current research and the literature. References include ref. 12, 20 and 50–52.

A comparative evaluation was conducted to assess the mechanical integrity of the tested composite laminates, focusing on tensile strength, demonstrating how the constituent elements and fabrication methods influence overall performance (Fig. 8). Both UD-S and UD-M laminates displayed outstanding tensile strength compared to data in the literature. 0.5 UD M laminate provides the maximum strength and stiffness in the load direction and is highly likely to be used for load-bearing applications such as Type V COPVs. However, twill configurations provide the lowest tensile properties among all the laminates tested and are ideal for use in multi-directional loading scenarios, where a slight reduction in mechanical strength is tolerable. The interplay between fibre architecture, resin type, and MXene integration is studied to support informed decisions based on desired strength, cost-effectiveness, and intended use. The unidirectional CFRP laminates displayed higher tensile properties compared to their weave counterparts, due to their highly oriented fibres, which ensure maximum load-transfer characteristics along the fibre direction. However, twill laminates showed progressive damage with improved damage tolerance, linked to their weave-like fibre pattern that provides interlacing points for load redistribution.

### 3.7. Fractured surface morphology

**Fig. 9:**
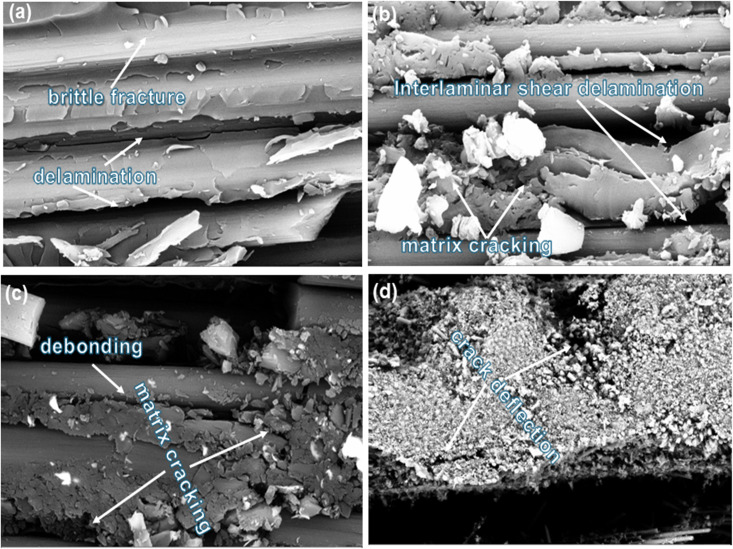
Fractured surface of tensile samples under SEM: (a) twill-S, (b) twill-M, (c) UD-S and (d) UD-M.

Following tensile testing, the fracture surfaces of twill, UD, and MXene-integrated specimens were examined under SEM to fully understand the root cause of failure (Fig. 9). The twill CFRP laminate (image a), displayed heterogeneous fractured surfaces. A rough surface can be seen due to the crimp and interlacing of fibres. In the case of MXene-integrated twill CFRP (image b), intricate crack-propagation trajectories and an overall enhancement in the matrix deformation were revealed. MXene contributed well to the attenuation of stress concentration at fibre interfaces and crimp-induced irregularities, facilitating a more complex failure mode. The rougher fractured surface confirmed that the integration of MXene resisted crack initiation and growth.

In UD-S CFRP laminates (image c), the fractured surface was associated with brittle fracture and sharp fibre breakage along the direction of the load. This type of morphology demonstrates a brittle failure with no evident plastic deformation. The evidence of fibre pullout in certain regions indicates diminished interfacial shear strength. The void formation adjacent to the fibre matrix debonding highlights poor interfacial stress transfer.

Interlaminar separation – either flat or stepped – was observed due to excess resin or insufficient compaction during fabrication. MXene-integrated UD CFRP laminates (image d) revealed a roughened and densely textured morphology, suggestive of improved interfacial bonding and energy absorption during crack growth. The fractured surface revealed a non-uniform surface texture due to the presence of MXene agglomerations. The structurally consolidated granular regions indicate strong interfacial bonding, where the MXene network potentially supports load distribution at the fibre-matrix interface. Unlike UD-S, the UD-M laminates displayed a more cohesive matrix failure mode, with rare signs of fibre debonding. The fragmented appearance of the epoxy resin and fine particulate structure promoted the role of MXene in suppressing crack growth by absorbing more energy at the crack tip. Taken collectively, the morphological studies confirmed that MXene-integrated UD CFRP laminates improved toughness and damage tolerance, shifting the failure mechanism from brittle to progressive fracture response entailing matrix shear and enhanced interfacial adhesion.

**Fig. 10:**
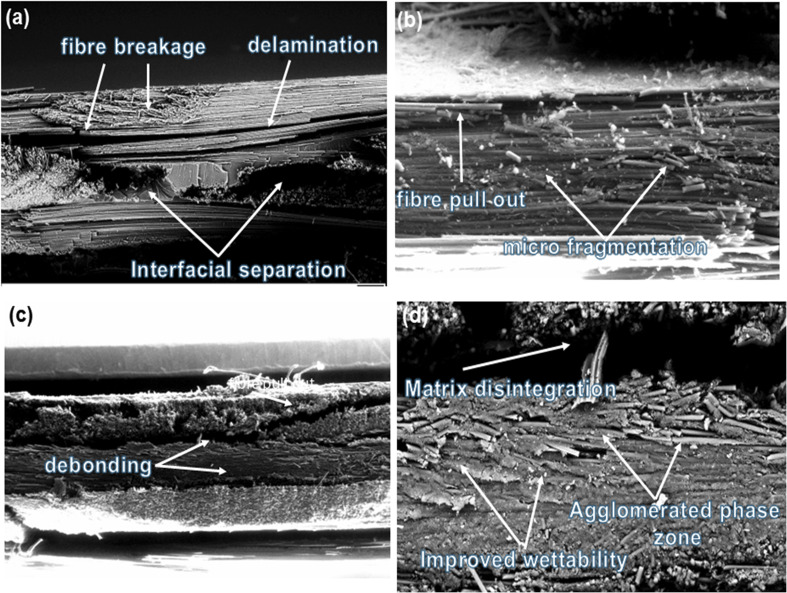
Interpretation of SEM images of CFRP (a) twill-S, (b) twill-M, (c) UD-S and (d) UD-M.

Fig. 10 displays the SEM fractography of the CFRP laminates after tensile testing. The twill-S sample (image a) displayed fragmented fibre bundles that were oriented in a [0,90] weave.

The failure resulted from the integration of fibre breakage, delamination, and matrix cracking. The central to lower region of the micrograph displays flat interlaminar separation, evidence of delamination or interfacial separation between adjacent plies. This damage is initiated by interlaminar shear stresses that become increasingly evident at the weave cross-over regions.

The twill-M samples (image b) displays numerous fibre fractures with disintegrated matrix areas, indicating a synergistic failure mechanism resulting from the combined effect of both fibre breakage and matrix cracking. The fibres appear tightly interlocked in epoxy resin, with no signs of fibre pull-out, suggesting enhanced adhesion between fibre and matrix due to MXene integration. The presence of dispersed micro-fragments and discontinuous fracture features satisfies the mixed mode failure, featuring both tensile failure and interlaminar shear delamination suppressed with MXene aspect ratio and structural efficiency.

In the UD-S samples (image c), the fractured surface displays a mix of matrix cracking and fibre pull-out, where carbon fibres extend irregularly beyond the matrix surface. This fracture is a clear indication of a mixed-mode failure with the combined effect of fibre breakage and debonding co-occurring. The fibres pulled out at non-uniform lengths, confirming non-uniform stress distribution during tensile testing of the UD carbon fibres.

In the UD-M samples (image d), the fractured surface reveals more complicated morphology, with larger matrix disintegration and uneven fibre-matrix interfaces. Carbon fibres pull out at varying lengths, with MXene-enriched resin still attached to their surface, pointing towards a shift in interfacial adhesion. The complex crack-growth patterns compared to UD-S indicate that the integration of MXene improved the fracture toughness, crack growth, and deflection approach. The fibre surface showed mixed traits, with enhanced matrix adhesion as the major trait due to MXene-improved interfacial bonding, while fibre pull-out was observed as a minor trait, signalling the prospect of MXene agglomeration that undermined the fibre-resin adhesion.

### 3.8. Hydrogen permeation measurement

Hydrogen permeation experiments through standard UD samples and MXene-integrated samples were conducted at different pressures, starting from one bar and reaching up to 5 bar, to examine where the specimens displayed gas permeation. A total of nine cycles were conducted. The standard UD specimens exhibited leakage at low pressures, which prevented reliable permeation data from being recorded using the experimental setup. This behaviour indicates insufficient barrier performance of the baseline composite under the tested conditions.

**Fig. 11:**
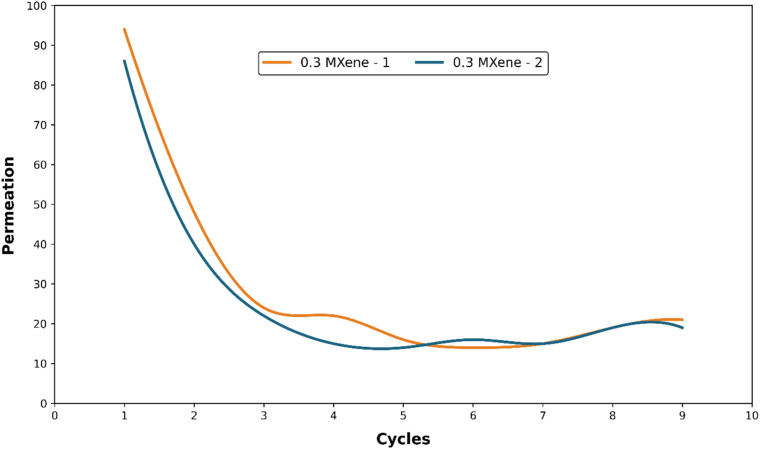
Hydrogen permeability through the thickness of the UD CFRP samples loaded at 0.3 wt% of MXene nanoflakes.

Both MXene-integrated samples at 0.3 wt% and 0.5 wt% displayed low permeability up to a pressure of 3 bar; however, at a higher pressure of 5 bar, they exhibited a distinct three-stage cycle-dependent permeation behaviour. Fig. 11 shows the permeability over a number of hydrogen exposure cycles for the two UD laminates enhanced with 0.3 wt% MXene. This repeated hydrogen exposure reveals important findings regarding the laminate's internal structure. Initially, both samples displayed a high permeation of 85 and 93 mL m^−2^ day^−1^ atm^−1^, followed by a sharp dip in values after the third exposure cycle. This initial declining behaviour reveals the transport behaviour triggered by high pressure, which has been extensively documented in the available literature on polymer membranes and gas barrier materials.^53,54^ High earlier hydrogen permeability could be attributed to the presence of micro-voids or poor interfacial adhesion, which mutually aided higher diffusion rates. Upon successive pressurization events, the polymeric molecular chains rearrange themselves and convert into a dense structure with a reduction in irregularities, leading to lower permeation rates.

**Fig. 12:**
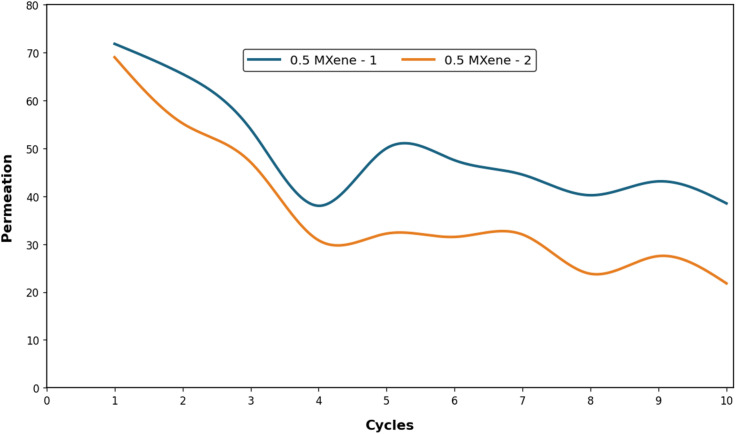
Hydrogen permeability through the thickness of the UD CFRP samples loaded at 0.5 wt% of MXene nanoflakes.

Both samples reached an equilibrium state between the fourth and seventh pressure cycle, with minor fluctuations in permeation rate. The laminates' internal morphology was characterized by a drop in void content and improved interfacial properties, which contribute to the formation of more tortuous pathways for hydrogen diffusion, hence creating a barrier to gas transport and validating the laminates' intrinsic barrier properties.^21,44^ This type of laminate behaviour is fundamental in addressing its reliability in real-life scenarios. A slight increase in permeation is observed between the 7th and 9th cycle, which may suggest the initiation of microstructural damage due to repeated high-pressure events. This could also promote the formation of micro-cracks, weaker interfaces, and slower slackening of molecular chains.^55^ The compaction effect of the samples is reversed over repeated pressurized events, resulting in the appearance of new diffusion channels with a trivial surge in the hydrogen permeation. A steady-state diffusion and sorption stage was attained after repeated pressurization events, as the values displayed marginal variations and appeared to converge. The converged region exhibited low permeation values of 18.82 mL m^−2^ day^−1^ atm^−1^ and 18.20 mL m^−2^ day^−1^ atm^−1^ for both samples loaded at 0.3 wt% of MXene, which were obtained by taking the average of the stabilized region values. Collectively, the permeation behaviour displayed by MXene-integrated laminates indicates conversion from a densely compact structure to structural breakdown, presenting deep insights into their sustainability. Fig. 12 displays the hydrogen permeation through UD samples loaded at 0.5 wt% of MXene; both highlight an initial sharp drop in permeability for the first four pressurizing cycles, followed by a steady state from the fifth to the tenth cycles. The decline in permeation is associated with transient controlled sorption diffusion. This kind of transition is commonly observed in polymeric composites that are repeatedly exposed to pressurized hydrogen gas, where a concentration gradient is developed across the sample thickness. A sharp dip in the permeation values up to the fourth cycle indicates the formation of tortuous diffusion channels due to lower defect concentration. During the second phase, a slight increment in values is displayed by sample 1 (0.5 wt% MXene-1), which is indicative of microstructural relaxation, which has paramount importance in linerless pressure vessels where the laminate itself acts as the hydrogen gas barrier. The second sample (0.5 wt% MXene-2) highlights a stable profile with the least relaxation effects. The characteristics displayed by the MXene-integrated composite samples align well with the reported literature on composite systems exposed to pressurized hydrogen, where optimal adhesion between the fibre and resin with a minimal number of fabrication defects promotes higher permeability under various loading scenarios.^51^ Also, the integration of nanoparticles into the matrix system helps in permeation rate reduction due to restricted molecular chain movements and constrained diffusion channels.^56^

As the successive pressurization events continue, both samples attain an equilibrium stage with insignificant wavering, but the permeability values continue to decline. The values converge after the fifth cycle, which is critical for lightweight hydrogen storage applications because it confirms that the composite system has reached a stable diffusion state, displaying sustainability. The lower hydrogen permeation shown by each sample confirms the formation of a structurally strong and tough microstructure, which is mandatory for Type V vessels. However, the sample loaded at 0.3 wt% displays lower permeation values relative to the 0.5 wt% sample due to lower manufacturing-induced irregularities. Additionally, the permeation results confirm that higher MXene loading at 0.5 wt% is still within the percolation threshold, as no nanoflake agglomeration is evident, which significantly lowers the overall performance of the composite system.

However, as a comparative screening approach for hydrogen permeation, the transition from premature leakage in standard UD laminates to a diffusion-controlled phenomenon in MXene-incorporated laminates demonstrates their capability to improve mechanical strength and offer an effective barrier to gas leakage, making them suitable for Type V COPV fabrication, provided that manufacturing-induced defects are minimized.

### 3.9. Fault analysis and remediation

**Fig. 13:**
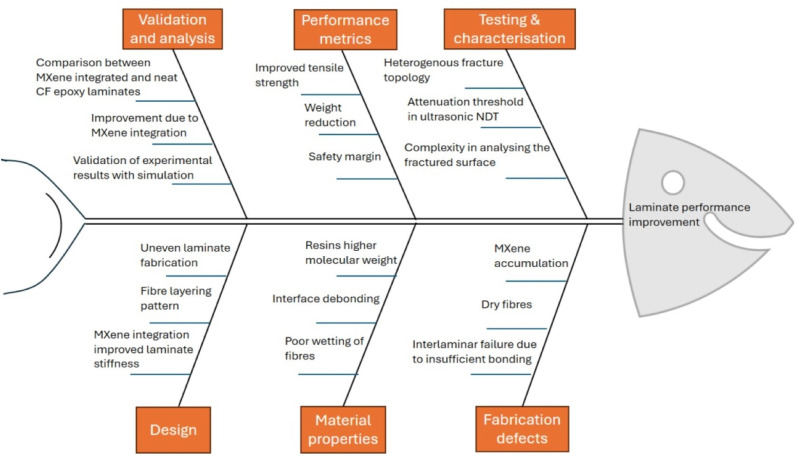
Fishbone diagram outlining the key factors influencing composite performance and failure.

This research indicates several failure mechanisms that can reduce the mechanical integrity of the composite laminate. These include MXene accumulations, dry fibres, matrix cracking, debonding, and fibre breakage (Fig. 13). The Ishikawa approach further categorized the contributing parameters into six primary domains: design, material properties, fabrication defects, testing and characterisation, performance metrics, and validation and analysis. Each domain was segmented to uncover the respective root causes based on experimental observations and pre- and post-failure analysis. While the incorporation of MXene nanoparticles has displayed potential for improving the tensile properties and thermal performance of the carbon-fibre-reinforced polymer laminates, inhomogeneous dispersion of MXene was observed during characterisation. MXene possess a high surface area to volume ratio due to their sub-atomic size, enabling the manifestation of unique physicochemical properties not found in the bulk materials. These attributes allow MXene-induced materials to form dense, uniform layers that effectively block corrosive agents and reduce gas permeation, thereby improving the material performance.^52^

In the design branch, the predominant issue stemmed from variability in laminate fabrication, the fibre layer pattern, and resin accumulations. The simulation results revealed that stress was more localized near the resin-dominant interlaminar zones, which correlate with early crack propagation in the tensile sample. Additionally, the layer thickness and fibre orientation significantly affected the stress distribution across the composite. In the material selection part, the underlying problem was driven by the resin's higher viscosity, inadequate surface treatment, and poor wetting of the fibres. The manufacturing process branch showcased process-induced defects, such as air entrapment, voids, and uneven curing, which were identified through NDT testing. MXene accumulations were also observed under SEM. These types of defects are most often found in laminates with non-ideal curing or inconsistent vacuuming. The mechanical integrity relied on tensile testing as a primary metric, which helps to predict the hoop stresses in Type V COPV. Standard CFRP laminates displayed linear behaviour, as expected, with brittle failure; however, the MXene-integrated laminates revealed a toughening effect. These deviations were strongly correlated with the presence of defects and non-uniform MXene integration. Through the structured lens of the Ishikawa analysis, the leading contributors to variability are highlighted as:

• Non-uniform dispersion of MXene nanoflakes.

• Epoxy pockets with weak interfacial bonding.

• Incomplete curing or vacuuming.

• Fabrication defects like voids, dry fibres or matrix cracking.

Each of these factors was interlinked, reinforcing the complex, multiscale nature of composite performance. The fishbone diagram not only maps these interactions but also helps prioritise corrective strategies such as optimising the mixing protocol for MXene dispersion, enhancing vacuum bagging control, and refining the cure cycle based on DSC data.

The fabrication of MXene-integrated UD carbon fibre laminates is proposed for applications in linerless Type V COPVs as the principal load-carrying structural shield.

The laminates can be incorporated into filament winding equipment or automated fibre placement as layered structural plies, within hoop-governed zones where maximum tensile stresses arise under the influence of hydrogen pressurization. A significant rise in tensile strength, interfacial adhesion, and resistance to damage helps to enhance mechanical efficiency under cyclic high-pressure hydrogen loading. Additionally, MXene integration leads to a more tortuous diffusion route, enhancing the barrier to hydrogen permeation, which is essential for linerless tanks' safety. Collectively, the anticipated structure presents a multifunctional laminate system that is appropriate for scalable deployment in future lightweight hydrogen tanks.

## 4 Conclusion

This research offers a multifactorial investigation landscape into the mechanical integrity and failure mechanisms of carbon fibre/epoxy composites, fabricated with and without MXene integration. The key conclusions are as follows.

MXene-integrated UD laminates at 0.3 and 0.5 wt% loading demonstrated 10.84% and 18.39% increases in tensile strength, respectively, relative to the standard laminate; however, the modulus rise was inconsistent due to MXene agglomeration. By increasing the MXene concentration to a certain threshold, the mechanical properties of the laminate can be enhanced. MXene integration also enhanced the thermal endurance and interfacial bonding. However, the role of MXene in improving the structural integrity is notably affected by the microstructural uniformity, ease of dispersion, and fabrication standard. SEM results indicate that MXene agglomeration can act as stress hot spots, negating the toughening benefits. Additionally, thermal analysis using TGA and DSC pointed towards curing variability, linked to matrix-rich zones and interfacial defects. The obtained results were further corroborated by FEA simulations, which identified stress concentrations near the grips, conforming with the tensile fracture pattern. The formulation of a diffusion-dominated equilibrium permeation state under cyclic pressurisation represents Fickian diffusion-influenced passage through tortuous pathways, highlighting the reliability of the MXene-integrated composites for long-term hydrogen-storage applications. In Type V COPV's, the inhibition of early hydrogen escape is itself a significant performance indicator. Hence, failure of the standard UD laminates and persistent efficiency of MXene-integrated composites present important comparative perceptions for barrier functionality. The Ishikawa (fishbone diagram) based root cause analysis revealed the comprehensive mapping of complex interactions between material properties, fabrication defects, testing variability, and key design elements. Significant impediments to compromised performance include non-uniform dispersion of MXene and process-induced defects, such as air entrapment, voids, and dry fibres. The results offer a material-level basis for the fabrication of MXene-incorporated composite laminates for hydrogen-storage pressure vessels while demonstrating their considerable influence on fabrication quality and defect minimization. However, the approach must be validated under high pressure to translate their progress into real-world engineering applications.

By supporting the experimental findings with simulations, the present research presents a reliable method to uncover and resolve failure causes in advanced lightweight carbon fibre composite laminates enhanced with MXene. The research insights are poised to play a pivotal role in future optimisation strategies in the defect-free fabrication of high-performance lightweight Type V COPVs.

## Author contributions

Ayyaz A. Janjua: conceptualisation, FEA simulations, methodology, formal analysis, investigation, data curation, original draft writing, writing – review and editing, visualisation. Oludare Amos Solademi: FEA simulations, editing. Emmanuel Okechukwu Achukwu: formal analysis, investigation, data curation, writing – review and editing, visualisation. Nadimul Faisal: conceptualisation, investigation, resources, data curation, supervision, writing – review and editing, visualisation. Mohd Shahneel Saharudin: conceptualisation, methodology, investigation, resources, data curation, formal analysis, writing – review and editing, visualisation, supervision.

## Conflicts of interest

The authors declare that they have no competing financial interests or personal relationships that could have appeared to influence the work reported in this paper.

## Data Availability

The data will be made available on request.

## References

[cit1] Ono H., Nait-Ali A., Castagnet S. (2023). Damage evolution in unfilled EPDM during various types of repeated hydrogen high-pressure cycles. Int. J. Fract..

[cit2] Lin J., Li J., Jiang C., Chen X., Tang Z., Zeng Z. (2022). *et al.*, Theoretical and experimental investigation of circumferential guided waves in orthotropic annuli. Ultrasonics.

[cit3] ASTM , Standard Test Method for Determining Gas Permeability Characteristics of Plastic Film and Sheeting, 1998

[cit4] Osman A. I., Chen L., Yang M., Msigwa G., Farghali M., Fawzy S. (2023). *et al.*, Cost, environmental impact, and resilience of renewable energy under a changing climate: a review. Environ. Chem. Lett..

[cit5] Slattery P. G., McCarthy C. T., O'Higgins R. M. (2016). Assessment of residual strength of repaired solid laminate composite materials through mechanical testing. Compos. Struct..

[cit6] Farooq S. A., Raina A., Mohan S., Arvind Singh R., Jayalakshmi S., Irfan Ul Haq M. (2022). Nanostructured coatings: Review on processing techniques, corrosion behaviour and tribological performance. Nanomaterials.

[cit7] Hassan I. A., Ramadan H. S., Saleh M. A., Hissel D. (2021). Hydrogen storage technologies for stationary and mobile applications: Review, analysis and perspectives. Renewable Sustainable Energy Rev..

[cit8] Iqbal K. , Development of a liner-less composite CNG cylinder and improved mechanical properties of cylinder materials, Doctoral dissertation, Hong Kong University of Science and Technology, 2008

[cit9] Jones B. H. and Li M. C. Liner-less tanks for space application-design and manufacturing considerations, In 5th conference on aerospace materials, processes, and environmental technology, 2003

[cit10] Fujiwara H., Ono H., Ohyama K., Kasai M., Kaneko F., Nishimura S. (2021). Hydrogen permeation under high pressure conditions and the destruction of exposed polyethylene-property of polymeric materials for high-pressure hydrogen devices (2)-. Int. J. Hydrogen Energy.

[cit11] Ab Ghani A. F., Mahmud J. (2018). Hardness, tensile and microstructure assessment of carbon/glass hybrid composite laminate. J. Mech. Eng..

[cit12] Quanjin M., Salim M., Rejab M., Bernhardi O. E., Nasution A. Y. (2020). Quasi-static crushing response of square hybrid carbon/aramid tube for automotive crash box application. Mater. Today: Proc..

[cit13] Katsivalis I., Signorini V., Ohlsson F., Langhammer C., Minelli M., Asp L. E. (2024). Hydrogen permeability of thin-ply composites after mechanical loading. Composites, Part A.

[cit14] Zhou W., zheng Z. W., Zhang Y. nan, Ding Z. jun (2018). Cluster analysis of acoustic emission signals and deformation measurement for delaminated glass fiber epoxy composites. Compos. Struct..

[cit15] Hassan A., Ilyas S. Z., Jalil A., Ullah Z. (2021). Monetization of the environmental damage caused by fossil fuels. Environ. Sci. Pollut. Res..

[cit16] Fremmelev M. A., Ladpli P., Orlowitz E., Dervilis N., McGugan M., Branner K. (2023). A full-scale wind turbine blade monitoring campaign: Detection of damage initiation and progression using medium-frequency active vibrations. Struct. Health Monit..

[cit17] Khan I., Zakari A., Zhang J., Dagar V., Singh S. (2022). A study of trilemma energy balance, clean energy transitions, and economic expansion in the midst of environmental sustainability: New insights from three trilemma leadership. Energy.

[cit18] Comba A., Scotti N., Maravić T., Mazzoni A., Carossa M., Breschi L. (2020). *et al.*, Vickers hardness and shrinkage stress evaluation of low and high viscosity bulk-fill resin composite. Polymers.

[cit19] Blanc-Vannet P. (2017). Burst pressure reduction of various thermoset composite pressure vessels after impact on the cylindrical part. Compos. Struct..

[cit20] Prasanthi P. P., Kumar M. N., Chowdary M. S., Madhav V. V., Saxena K. K., Mohammed K. A. (2023). *et al.*, Mechanical properties of carbon fiber reinforced with carbon nanotubes and graphene filled epoxy composites: experimental and numerical investigations. Mater. Res. Express.

[cit21] Gupta M., Lin Y., Deans T., Baer E., Hiltner A., Schiraldi D. A. (2010). Structure and gas barrier properties of poly (propylene-graft-maleic anhydride)/phosphate glass composites prepared by microlayer coextrusion. Macromolecules.

[cit22] Krishna R. , Titus E., Salimian M., Okhay O., Rajendran S. and Rajkumar A., *et al.*, Hydrogen Storage for Energy Application, Hydrogen Storage, 2012, 243

[cit23] Souza G., Tarpani J. R. (2021). Using OBR for pressure monitoring and BVID detection in type IV composite overwrapped pressure vessels. J. Compos. Mater..

[cit24] Zhou W., Ji X. L., Yang S., Liu J., Ma L. H. (2021). Review on the performance improvements and non-destructive testing of patches repaired composites. Compos. Struct..

[cit25] Alam S., Yandek G. R., Lee R. C., Mabry J. M. (2020). Design and development of a filament wound composite overwrapped pressure vessel. Compos., Part C: Open Access.

[cit26] Haldar A. K., Zhou J., Guan Z. (2016). Energy absorbing characteristics of the composite contoured-core sandwich panels. Mater. Today Commun..

[cit27] Council H. , Path to hydrogen competitiveness: A cost perspective, A policy paper, 2020, https://www.h2knowledgecentre.com/content/policypaper1202?crawler=redirect&mimetype=application/pdf

[cit28] Romanello M., Di Napoli C., Drummond P., Green C., Kennard H., Lampard P., Costello A. (2022). The 2022 report of the Lancet Countdown on health and climate change: health at the mercy of fossil fuels. Lancet.

[cit29] Rejab M., Cantwell W. J. (2013). The mechanical behaviour of corrugated-core sandwich panels. Composites, Part B.

[cit30] Allen T., Ahmed S., Hepples W., Reed P. A., Sinclair I., Spearing M. (2018). A comparison of quasi-static indentation and low-velocity impact on composite overwrapped pressure vessels. J. Compos. Mater..

[cit31] Prabhune P., Chen A., Comlek Y., Chen W., Brinson L. C. (2025). Process-structure–property relation for elastoplastic behavior of polymer nanocomposites with agglomerates and interfacial gradients. Compos. Sci. Technol..

[cit32] Yampolskii Y. (2012). Polymeric gas separation membranes. Macromolecules.

[cit33] Figiel Ł., De Angelis M. G., Janssen F., Vehlow D., Giannis S., Skytree L. (2025). *et al.*, Polymers and composites for hydrogen economy: a perspective. J. Mater. Sci.: Compos..

[cit34] Alves M. L., Santana P., Fernandes N., Martins P. (2013). Fabrication of metallic liners for composite overwrapped pressure vessels. Int. J. Adv. Des. Manuf. Technol..

[cit35] Perillo G., Grytten F., Sørbø S., Delhaye V. (2015). Numerical/experimental impact events on filament wound composite pressure vessel. Composites, Part B.

[cit36] Curtis J., Hinton M. J., Li S., Reid S. R., Soden P. D. (2000). Damage, deformation and residual burst strength of filament-wound composite tubes subjected to impact or quasi-static indentation. Composites, Part B.

[cit37] Njuguna J. , Lightweight Composite Structures in Transport: Design, Manufacturing, Analysis and Performance, Woodhead publishing, 2016

[cit38] Murray B. R., Leen S. B., Semprimoschnig C. O., Brádaigh C. M. Ó. (2016). Helium permeability of polymer materials as liners for composite overwrapped pressure vessels. J. Appl. Polym. Sci..

[cit39] Reynolds J., Ali D., Njuguna J., Amadhe F. (2024). The state of the art in hydrogen storage. Green Energy Environ. Technol..

[cit40] Ray P., Yu X., Fan Z., Srinivasan B., Rajagopal P. (2019). Fiber bragg grating based detection of part-thickness cracks in bent composite laminates using feature-guided waves. Smart Mater. Struct..

[cit41] Rafiee R., Fakoor M., Hesamsadat H. (2015). The influence of production inconsistencies on the functional failure of GRP pipes. Steel Compos. Struct..

[cit42] ASTM A. M. , ASTM D3039-standard test method for tensile properties of polymer matrix composite materials, ASTM D3039-standard test method for tensile properties of polymer matrix composite materials, ASTM International, 2017, p. 360

[cit43] Chou H. Y., Mouritz A. P., Bannister M. K., Bunsell A. R. (2015). Acoustic emission analysis of composite pressure vessels under constant and cyclic pressure. Composites, Part A.

[cit44] Idris A., Muntean A., Mesic B. (2022). A review on predictive tortuosity models for composite films in gas barrier applications. J. Coat. Technol. Res..

[cit45] Satyapal S., Petrovic J., Read C., Thomas G., Ordaz G. (2007). The US Department of Energy's National Hydrogen Storage Project: Progress towards meeting hydrogen-powered vehicle requirements. Catal. Today.

[cit46] Opelt C. V., Becker D., Lepienski C. M., Coelho L. A. (2015). Reinforcement and toughening mechanisms in polymer nanocomposites–carbon nanotubes and aluminum oxide. Composites, Part B.

[cit47] Mallick K. , Cronin J., Arzberger S., Tupper M., Grimes-Ledesma L. and Lewis J., *et al.*, Ultralight linerless composite tanks for in-space applications, in Space 2004 Conference and Exhibit, 2004, p. 5801

[cit48] Zhang P. F., Zhou W., Yin H. F., Shang Y. J. (2019). Progressive damage analysis of three-dimensional braided composites under flexural load by micro-CT and acoustic emission. Compos. Struct..

[cit49] Ma Q., Rejab M., Hassan S. A., Azeem M., Saffirna M. S. (2023). Failure behavior analysis of the Spherical-Roof Contoured Core (SRCC) under quasi-static loading: A numerical study. J. Fail. Anal. Prev..

[cit50] Ibrahim M. I., Rejab M. R. M., Romli N. K., Quanjin M., Rani M. F. (2023). Effects on hybridization of interlayer composites and self-reinforced polypropylene. J. Mech. Eng. Sci..

[cit51] Ekşi S., Genel K. (2017). Comparison of mechanical properties of unidirectional and woven carbon, glass and aramid fiber reinforced epoxy composites. Acta Phys. Pol., A.

[cit52] Sapuan S. M., Aulia H. S., Ilyas R. A., Atiqah A., Dele-Afolabi T. T., Nurazzi M. N. (2020). *et al.*, Mechanical properties of longitudinal basalt/woven-glass-fiber-reinforced unsaturated polyester-resin hybrid composites. Polymers.

[cit53] Rivard E., Trudeau M., Zaghib K. (2019). Hydrogen storage for mobility: a review. Materials.

[cit54] Vasiliev V. V. , Composite Pressure Vessels: Design, Analysis, and Manufacturing, Bull Ridge Corporation, 2009

[cit55] Ma X., Zare Y., Rhee K. Y. (2017). A two-step methodology to study the influence of aggregation/agglomeration of nanoparticles on Young’s modulus of polymer nanocomposites. Nanoscale Res. Lett..

[cit56] Ryan K. , Cronin J., Arzberger S., Mallick K., Munshi N., Yazdani F., *et al.*, Prediction of pressure cycle induced microcrack damage in linerless composite tanks, in 47th AIAA/ASME/ASCE/AHS/ASC Structures, Structural Dynamics, and Materials Conference, 2006, p. 2201

